# Corrective exercises strongly improve posture but fail to produce consistent clinical or functional benefits in patients with upper crossed syndrome: a systematic review and meta-analysis of randomized controlled trials

**DOI:** 10.1186/s13102-026-01707-8

**Published:** 2026-05-28

**Authors:** Fateme Khorramroo, Mohamad Rostami, Mostafa Jalili Bafrouei

**Affiliations:** 1 Department of Sport Injuries and Biomechanics, Faculty of Sport Sciences and Health, University of Tehran, Tehran, Iran; 2 Monash Exercise Neuroplasticity Research Unit, Department of Physiotherapy, School of Primary and Allied Care, Faculty of Medicine, Nursing and Health Science, Monash University, Melbourne, Australia

**Keywords:** Kyphosis, Round Shoulder, Forward Head, Therapeutic, Rehabilitation

## Abstract

**Background:**

Upper Crossed Syndrome (UCS) is a prevalent postural dysfunction involving muscular imbalances and misalignments of the neck, thoracic spine, and shoulder girdle, leading to pain and functional limitation. While corrective exercises (CE) are widely recommended to restore posture and improve musculoskeletal function, their effectiveness across different outcomes remains unclear. This systematic review and meta-analysis evaluated the effects of CE on posture, function, and pain in individuals with UCS.

**Methods:**

Four databases (PubMed, Web of Science, Scopus, Embase) were searched from inception to November 2025. Eligibility criteria included randomized controlled trials (RCTs) on individuals with UCS evaluating CE versus controls. Data extraction followed the PRISMA 2020 guidelines. Risk of bias was assessed using the RoB 2 tool. Random-effects models were used to calculate standardized mean differences (SMD) with 95% confidence intervals (CI). Heterogeneity (I^2^), publication bias, and subgroup effects by age and exercise type were evaluated, and evidence certainty was rated using the GRADE approach.

**Results:**

Twenty-eight RCTs (*n* = 901) were included. CE significantly improved forward head angle (SMD: -1.49; 95% CI: -1.91 to -1.06; *p* < 0.01) with high heterogeneity (I^2^ = 79%, *p* < 0.01), forward shoulders angle (SMD: -1.53; 95% CI: -1.96 to -1.09; *p* < 0.01) with high heterogeneity (I^2^ = 78%, *p* < 0.01), and thoracic hyper-kyphosis angle (SMD: -1.70; 95% CI: -2.27 to -1.12; *p* < 0.01) with high heterogeneity (I^2^ = 88%, *p* < 0.01). However, the effects on muscle activation, function, balance, and pain were inconclusive.

**Conclusion:**

CE produces large, significant improvements in postural alignment in individuals with UCS. However, evidence for clinically meaningful benefits in pain, function, and muscle strength remains limited and inconsistent. These findings suggest that while CE effectively addresses biomechanical postural deficits, their translation to symptom relief and functional improvement is uncertain. Future research should establish standardized CE protocols, include long-term follow-up, and employ validated clinical outcome measures to determine whether postural corrections yield sustained clinical benefits.

**Supplementary Information:**

The online version contains supplementary material available at 10.1186/s13102-026-01707-8.

## Introduction

Upper crossed syndrome (UCS) is a common postural disorder involving muscular imbalances and joint dysfunctions in the cervicothoracic region and shoulder girdle, particularly the glenohumeral, atlanto-occipital, and thoracic joints [1]. Rather than a simple imbalance of tight and weak muscles, UCS represents a crossed-pattern dysfunction marked by stiffness and inhibition across the anterior and posterior muscle groups [2], leading to disrupted length-tension relationships and impaired force control [3]. Prevalence estimates range widely from 11 to 60%, depending on occupation and population characteristics, with higher rates among office workers and students [4].

Occupational habits, altered proprioceptive input, and psychological factors, including low self-esteem or depressive symptoms, may contribute to UCS development [5]. These disruptions may impair sensorimotor control and scapular activation, compromising postural alignment and 3-dimensional coordination [6]. Consequently, the scapula may shift into abduction, internal rotation, anterior tilt, and protraction, promoting forward head posture, forward shoulders, thoracic hyper-kyphosis, and impaired static and dynamic balance [7]**.** These postural deviations may produce mechanical stress, resulting in pain, restricted mobility, limited ribcage expansion, and progressive imbalance [8] and may trigger distal dysfunctions such as lumbar hyper-lordosis, affecting the back and anterior thigh muscles' recruitment patterns [9].

Corrective exercise (CE) programs have emerged as a primary intervention to address UCS and prevent secondary complications. Various interventions have been studied, including aquatic exercises, strength training, stretching routines, National Academy of Sports Medicine (NASM-based) protocols, and comprehensive programs [7]. While preliminary evidence suggests therapeutic exercise may improve postural alignment and reduce symptoms [7, 9], current evidence remains fragmented and inconsistent, with studies reporting heterogeneous effects across key outcomes, including pain intensity [10, 11], muscle activity patterns [11, 12], balance performance, and functional measures [13]. No standardized CE protocol exists, and the effectiveness of CE across different outcome domains, particularly postural alignment, pain, and functional capacity, remains unclear. This lack of synthesis prevents clinicians from making evidence-based decisions about optimal CE prescription for UCS.

To address this gap, we conducted a systematic review and meta-analysis to synthesize current evidence on CE for UCS. The primary objective was to evaluate the effects of CE on postural parameters (forward head, forward shoulders, and thoracic hyper-kyphosis angles) in individuals with UCS. Secondary objectives included assessing effects on pain, functional capacity, muscle strength, and balance. We hypothesized that CE would significantly enhance alignment, improve faulty muscle activation, restore function, balance, and decrease pain in this population.

## Method

This systematic review adhered to the Preferred Reporting Items for Systematic Reviews and Meta-Analysis PRISMA guidelines [14]. The objective was to identify, evaluate, and synthesize studies that examined posture, pain, and function of the CE in individuals with UCS. The protocol was registered in PROSPERO (CRD420250653442).

### Search strategy

We identified the relevant studies through four electronic databases: PubMed, Web of Science, Scopus, and Embase. The search was run on November 18, 2025. The databases were interrogated with keywords following the PICO framework.

Key terms used in the search strategy were based on broad terms and related synonyms targeting 3 categories: #1 "upper crossed syndrome" OR “upper-crossed syndrome” OR UCS #2 "corrective exercise*" OR “corrective approach*” OR "National Academy of Sports Medicine" OR “NASM” #3 “Forward head” OR “forward shoulder” OR hyperkyphosis OR “thoracic kyphosis” OR "muscle activ*" OR "activation of muscle" OR "muscle electromyography" OR "muscle-EMG" OR balance OR pain OR function* OR performance #4 (1 AND 2 AND 3).

Additionally, Google Scholar reference lists from previous systematic reviews on UCS in UCS populations were manually searched to ensure the inclusion of all relevant studies.

### Eligibility criteria

Studies were included based on the following PICO criteria:

#### Population

Adolescents (8–18 years) and adults (19–45 years), athletes or non-athletes, defined by at least one of the following: forward head angle > 46°, forward shoulder angle > 52°, or thoracic kyphosis > 42°, who had been diagnosed with UCS [9].

#### Intervention

Any CE program informed by the principles of Florence Kendall or Shirley Sahrmann, including (but not limited to) NASM-based protocols, eccentric, concentric, isometric, resistance, and strengthening exercises aimed at improving forward head angle, forward shoulder angle, and thoracic hyper-kyphosis, as well as pain and functional outcomes. This also included exercises targeting the cervical and upper thoracic regions either directly or indirectly through multi-joint or upper-limb corrective approaches.

#### Comparators

Control conditions varied across studies and were categorized as follows: (1) continuation of regular athletic training in athlete populations, (2) passive controls including no-intervention or usual daily activities, and (3) alternative condition such as vestibular exercise or health education programs with UCS.

#### Outcomes

Primary outcomes included posture-related measures assessed using side photography [15], photogrammetry [15], and a flexible ruler [16], which have demonstrated good-to-excellent intra- and inter-rater reliability and acceptable validity for assessing forward head, forward shoulder, and thoracic kyphosis angles. Secondary outcomes included objective and self-reported assessments of pain using the visual analog scale (VAS) [17], which has high test–retest reliability and well-established construct validity, and a pain ruler [18], which has demonstrated acceptable reliability and validity. Functional performance measures included range of motion (ROM) assessed by a goniometer, with high intra- and inter-rater reliability and acceptable to good criterion validity [19]; muscle activation assessed by electromyography (EMG), which is widely used and considered reliable for evaluating neuromuscular activation when standardized procedures are applied [20]; and dynamic and static balance assessed using the Y-Balance Test, which shows good-to-excellent reliability and acceptable validity [21], and the Biodex Balance System, which demonstrates high reliability and acceptable construct validity for balance assessment [22].

#### Study design

Randomized controlled trials (RCTs).

#### Language and publication status

Peer-reviewed publications (at least abstracts) in English.

The exclusion criteria comprised studies involving participants without a confirmed diagnosis of the UCS; interventions that did not include CE, informed by the principles of Florence Kendall or Shirley Sahrmann; and protocol studies, non-randomized study designs, including case studies, observational studies, and cohort studies. Publications who do not have at least their abstracts in English were excluded. Additionally, conference abstracts, letters, and editorials lacking full methodological details were omitted.

All records were first imported into EndNote, Version 21, reference management software for deduplication and screening. Following study selection, the included studies were imported into the Comprehensive Meta-Analysis software (CMA, Version 4) for data analysis. FK and MJB independently screened the titles and abstracts to identify relevant studies. A calibration exercise was conducted on a random subset of studies before full screening to ensure consistency. Inter-rater reliability during both title/abstract screening and full-text review was quantified using Cohen’s kappa (κ), with κ ≥ 0.80 interpreted as strong agreement. Disagreements were resolved by consensus or by consulting the reviewer, MR. Any missing or incomplete data in the included studies were addressed by contacting the corresponding authors when possible. If sufficient data were not provided, the conference study was excluded from the analysis.

### Data extraction

FK and MJB independently collected information from the included studies using a standardized extraction form to ensure consistency. Agreement between reviewers was quantified using Cohen’s kappa (κ), with κ ≥ 0.75 set a priori as acceptable reliability. MR resolved discrepancies. The following information was recorded: author and year, study design, participants’ demographics (age, sex, height, weight), CE protocol, tools, intervention and duration, inclusion and exclusion criteria, funding information, and outcome measures, including forward head posture, forward shoulder posture, thoracic kyphosis, muscle activation, function, balance, and pain.

### Quality assessment

Methodological quality was appraised using the PEDro checklist [17]. Scores were interpreted as: poor (< 4), fair (4–5), good (6–8), or excellent (9–10) [18]. FK and MJB assessed quality independently. Inter-rater reliability was quantified using weighted Cohen’s kappa, interpreted according to Landis and Koch (κ < 0.20 = poor; 0.21–0.40 = fair; 0.41–0.60 = moderate; 0.61–0.80 = substantial; > 0.80 = almost perfect agreement). Discrepancies were resolved by consensus. 

### Synthesis of result

Standard Mean differences (SMDs) and 95% confidence intervals (CI) were calculated using a random-effects model instead of a fixed-effects model in the CMA due to expected heterogeneity in study designs, tools, and outcome measures. Meta-analyses were performed when two or more studies evaluated the same outcome measure using similar methodologies. Statistical heterogeneity of the combined data was assessed using I^2^ statistics and corresponding P-values (*P* < 0.05). Publication bias was assessed at the overall level for outcomes with ≥ 10 studies using funnel plots and Egger’s regression test (*p* > 0.05 indicating no significant asymmetry); it was not evaluated for subgroups or outcomes with < 10 studies due to low power [23]. When detected, the trim-and-fill method estimated its impact on meta-analytic results and the number of potentially missing studies.

Meta-regression was performed using a random-effects model to assess whether intervention duration moderated the pooled effect size, but only for outcomes with ≥ 10 studies and sufficient between-study heterogeneity; outcomes with fewer studies or limited variability were not analyzed due to low statistical power and methodological limitations [24]. The results were interpreted based on the levels of evidence established by Tulder et al. [25] and modified by Jalili-Bafrouei, which was used to categorize the strength of evidence in Table 1. The quality of evidence for each outcome was evaluated using the GRADE (Grading of Recommendations, Assessment, Development, and Evaluation) framework, considering risk of bias, inconsistency, indirectness, imprecision, and publication bias. Evidence certainty was classified as high, moderate, low, or very low [26].

**Table 1:** Definitions of modified level of evidence by Jalili Bafrouei

Level of evidence	Description
Strong evidence	Pooled results from three or more studies, including a minimum of two high-quality studies, such as the PEDro scale for RCTs, the Newcastle–Ottawa scale (NOS) for non-RCTs, and which are statistically homogenous (*p* > 0.05), or may be associated with statistically significant or non-significant pooled results. An I^2^ cut-off to aid interpretability (I^2^ < 50% = low, 50–74% = medium, > 75% = high)
Moderate evidence	Statistically significant pooled results from multiple studies, including at least one high-quality study, which are statistically heterogeneous (*p* < 0.05); or from multiple low- or moderate-quality studies which are statistically homogenous (*p* > 0.05); or statistically insignificant pooled results from multiple studies, including at least one high-quality study, which are statistically homogenous (*p* > 0.05). An I^2^ cut-off to aid interpretability (I^2^ < 50% = low, 50–74% = medium, > 75% = high)
Limited evidence	Results from multiple low- or moderate-quality studies, which are statistically heterogeneous (*p* < 0.05), or from one high-quality study. An I^2^ cut-off to aid interpretability (I^2^ < 50% = low, 50–74% = medium, > 75% = high)
Very limited evidence	Results from one low- or moderate-quality study
Conflicting evidence	Pooled results that are insignificant and from multiple studies, regardless of quality, which are statistically heterogeneous (*p* < 0.05, i.e., inconsistent)

## Results

### Study selection

**Fig. 1:**
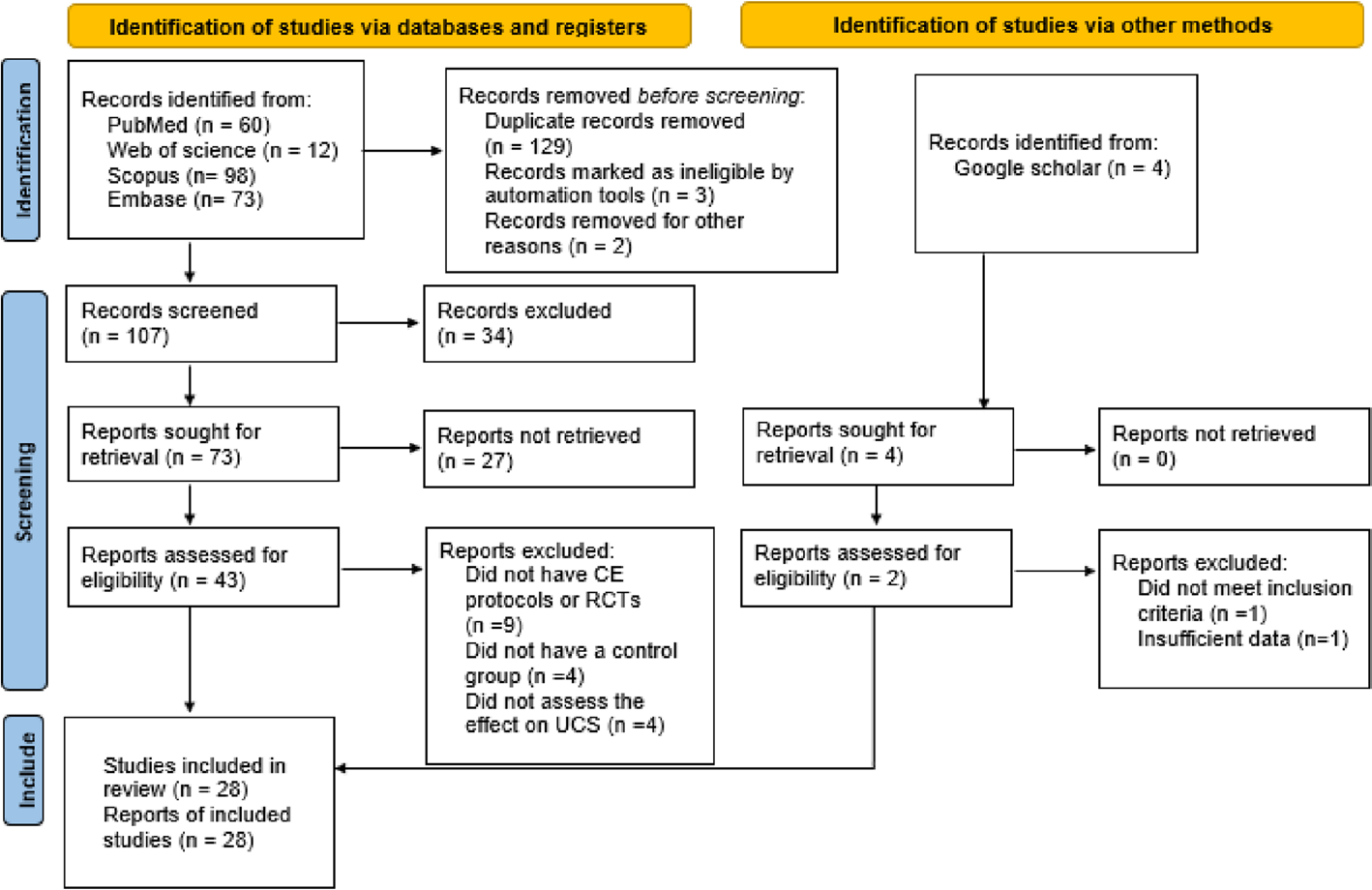
Flow diagram of the selection process

The main literature search yielded a total of 241 items: PubMed (60) studies, Web of Science (12), Scopus (98), and Embase (73) items, from which 112 items remained after duplicate removal. After screening the titles and abstracts, 28 studies were included and assessed. Figure 1 shows the flow diagram of the selection process and the number of excluded studies at each stage.

### Study characteristics

Table 2 summarizes the characteristics of the included studies. Publication years ranged from 2018 to 2025 (median = 2022), with 64.28% (18/28) of trials published after 2022. A total of 28 randomized controlled trials involving 901 participants were included in this systematic review. All included studies [10–13, 27–50] underwent rigorous methodological quality assessment using standardized appraisal tools. The trials were conducted primarily in adolescents and young adults, with participant ages spanning from early adolescence to middle adulthood and sample sizes typically ranging from 23 to 40 participants per study. Most interventions consisted of CE-based programs delivered over 6–12 weeks, commonly performed three sessions per week, with session durations between 20 and 70 min. The experimental interventions were predominantly NASM-based CE protocols or strengthening-oriented CE programs, while control conditions varied across studies. In most studies involving non-athlete participants, control groups received no exercise intervention or continued usual daily activities. In four studies involving athlete populations, the control groups continued their routine sport training (e.g., swimming, volleyball, basketball, and handball), representing maintenance of usual athletic activity rather than a structured intervention.

**Table 2:** Study and experiment characteristics of studies investigating the effects of CE on Upper Crossed Syndrome

Authors	Study design	Participants	Duration	Intervention	CE protocol	Outcomes, Tools & Brand	Age (year), Sample size, and sex	Anthropometry	Inclusion criteria	Exclusion criteria
Abdolahzadeh et al. 2019 [27]	RCT	UCS	8 Weeks, 3 per week, 30–70 min	Experimental group (EG): NASM CE, Control group (CG): Usual daily activity	Framework: NASM CE model, structure: warm-up (5–10 min), corrective strengthening and stretching (20–40 min), cool-down (5–10 min), soft-tissue release (cervical and upper shoulder muscles), stretching (chest, shoulder, cervical muscles), strengthening/activation (deep cervical flexors, scapular stabilizers), functional integration (postural and multi-joint exercises), progression: simple to complex, intensity: individualized based on fitness level and tolerance, rest intervals: based on set duration, supervision: supervised exercise sessions	Forward head posture (digital lateral photogrammetry, AutoCAD), forward shoulder posture (digital lateral photogrammetry, AutoCAD), Thoracic kyphosis (flexible ruler)	(Age: EG: 20.53 ± 1.55, CG: 20.00 ± 2.00), (30 females, EG:15, CG:15)	Height: EG: 160 ± 2, CG: 159 ± 30, Weight: EG: 60.53 ± 4.10, CG: 58.13 ± 5.08, BMI: EG:23.61 ± 1.20, CG: 22.71 ± 1.41	Forward head > 46 degrees, forward shoulder > 52°, and H1	Any related pathological symptoms, history of fractures, surgery, joint diseases, or failure to complete an exercise program
Aghaei et al. 2024 [13]	RCT	UCS	8 Weeks, 3 per week, 30–70 min	EG: NASM CE, CG: Vestibular exercise (Cawthorne-Cooksey)	Group 1: Cawthorne–Cooksey: sitting/supine/standing, eye and head movements (open/closed eyes), eye–hand coordination, sit-to-stand, object handling, ball throwing/catching, aim: vestibular balance training, Group 2: Postural CEs: supine/prone, pectoral stretching, deep neck flexor and core activation, scapular and shoulder exercises with bands, squat with gym ball, aim: posture correction	Dynamic stability (Y Balance Test), Forward head posture (lateral photography, Kinovea), forward shoulder posture (lateral photography, Kinovea), and thoracic kyphosis (flexible ruler)	(Age: 8.53 ± 0.74), (30 Male students EG: 15, CG: 15)	Height: 130.73 ± 7.20, Weight: 29.87 ± 8.04, BMI: NR	Abnormalities including forward head posture exceeding 46 degrees, kyphosis greater than 42 degrees, forward shoulders exceeding 52 degrees, and an age range of 8 to 10 years, no previous history of fractures or dislocations, no injuries or surgeries in the upper or lower joints, lack of participation in regular weekly activities, and overall physical health of the subjects	Non-participation in training programs, illness or inability to perform exercises, failure to participate in the second measurement phase, or lack of interest from the subject to participate in the research
Ahmadi et al. 2019 [30]	RCT	UCS	8 Weeks, 3 per week, 30–45 min	EG: NASM CE, CG: No intervention	warm-up 10–15 min, CE 25–35 min, cool-down 5–10 min, setting: water-based, framework: Janda CE approach, phases: inhibition (myofascial release and trigger point release in water, foam rolling), lengthening and activation (static stretching to restore muscle balance, breathing pattern correction for accessory respiratory muscles, strengthening for weak muscles), sensorimotor and proprioceptive training (cervical and shoulder region), integration/functional phase (maintaining correct posture during ball games in water), target: upper crossed syndrome correction	Pain (Visual Analog Scale), Neck muscle endurance (Neck Muscle Endurance Test), Dominant shoulder range of motion (flexion, extension, abduction, internal/external rotation, digital inclinometer, Acumar C405)	(EG: 22.42 ± 1.32, CG: 23.50 ± 0.8), (30 Male students, EG: 14, CG: 16)	Height: EG: 172.90 ± 1.10, CG: 170.60 ± 1.38, Weight: EG: 71.90 ± 2.6, CG: 70.62 ± 1.81, BMI: EG: 23.97 ± 0.77, CG: 23.22 ± 0.43	Forward head posture exceeding 46 degrees, kyphosis greater than 42 degrees, forward shoulders exceeding 52 degrees, neck or chest pain, and working with a laptop or smartphone for more than 6 h	History of competitive sports, professional sports, fractures, surgeries, respiratory problems, migraines, sensitivity to water, cardiac and pulmonary issues
Ahmadi et al. 2020 [28]	RCT	UCS	8 Weeks, 3 per week, 50–70 min	EG: Strengthening CE, CG: No intervention	warm-up 10–15 min, CE 25–35 min, cool-down 5–10 min, setting: water-based, framework: three-phase CE model, phases: inhibition (trigger point release via water massage and myofascial release using foam roller), lengthening and activation (static stretching for tight muscles, breathing pattern correction targeting accessory respiratory muscles in upper crossed syndrome, strengthening of weak muscles), sensorimotor and proprioceptive training (cervical and shoulder joints), integration/functional phase (postural control during ball games in water), target: muscular imbalance and upper crossed syndrome correction	Forward head and forward shoulder angles (Sony Cyber-shot DSC-RX100VI camera, AutoCAD 2013), Thoracic kyphosis (Staedtler Mars flexible ruler), Static and semi-dynamic balance (Biodex Balance System SD)	(Age: EG: 22.42 ± 1.32, CG: 23.50 ± 0.8), (30 Male students, EG: 14, CG: 16)	Height: EG: 172.90 ± 1.10, CG: 170.60 ± 1.38, Weight: EG: 71.90 ± 2.61, CG: 70.62 ± 1.81, BMI: EG: 23.97 ± 0.77, CG: 23.22 ± 0.43	Forward head posture exceeding 46 degrees, kyphosis greater than 42 degrees, forward shoulders exceeding 52 degrees, neck or chest pain, and working with a laptop or smartphone for more than 6 h	History of competitive sports, professional sports, fractures, surgeries, respiratory problems, migraines, sensitivity to water, cardiac and pulmonary issues
Ahmadi et al. 2022 [29]	RCT	UCS	8 Weeks, 3 per week + E3, 50–70 min	EG: Strengthening CE, CG: No intervention	warm-up 10–15 min, CE 25–35 min, cool-down 5–10 min, setting: water-based, framework: three-phase CE model (Janda), phases: inhibition (trigger point and myofascial release via water massage and foam roller), lengthening and activation (static stretching for tight muscles, breathing pattern correction for accessory respiratory muscles in upper crossed syndrome, strengthening of weak muscles), sensorimotor and proprioceptive training (cervical and shoulder joints), integration/functional phase (maintaining correct posture during ball games in water), target: muscular imbalance and upper crossed syndrome correction	Forward head and forward shoulder angles (lateral photographs, Sony Cyber-shot DSC-RX100VI, AutoCAD 2013), Thoracic kyphosis (flexible ruler)	(Age: EG: 22.42 ± 1.32, CG: 23.50 ± 0.8), (30 Male students, EG: 14, CG: 16)	Height: EG: 172.90 ± 1.10, CG: 170.60 ± 1.38, Weight: EG: 71.90 ± 2.61, CG: 70.62 ± 1.81, BMI: EG: 23.97 ± 0.77, CG: 23.22 ± 0.43	Forward head posture exceeding 46 degrees, kyphosis greater than 42 degrees, forward shoulders exceeding 52 degrees, neck or chest pain, and working with a laptop or smartphone for more than 6 h	History of competitive sports, professional sports, fractures, surgeries, respiratory problems, migraines, sensitivity to water, cardiac and pulmonary issues
Arshadi et al. 2019 [12]	RCT	UCS	8 Weeks, 3 per week, 50–70 min	EG: Strengthening CE, CG: No intervention	Supervised stretching, cervical stabilization, and scapular strengthening exercises with 5-min warm-up and cool-down. Stretching progressed from 30-s holds (+ 5 s every 2 weeks). Cervical stabilization (deep neck flexors/craniocervical joint) was performed supine and quadruped without load, progressing from 6 reps/2-s holds to 10 reps/10-s holds. Scapular strengthening loads were individualized (10RM), progressing from 40 to 70% 10RM over the intervention	Surface EMG (DataLog p3X8, Biometrics UK, analyzed with Datalink 5.06, on UT, MT, LT, SA, SCM, 1000 Hz)	(Age: EG: 21.44 ± 2.06, CG: 20.14 ± 1.71), 30 Males, EG: 12, CG: 13)	Height: EG: 174.2 ± 4, CG: 176.86 ± 4.7, Weight: NR, BMI: EG: 20.62 ± 3.9, CG: 21.20 ± 1.96	Craniocervical angle and forward shoulder—needed to be more than 46 and 52, respectively, thoracic. The kyphosis angle needed to be greater than 42	BMI greater than 29.9 kg/m2, having a history of surgery and fracture in the upper limb and spine during the past year, the presence of neurological disease, scoliosis, failing to attend pre- and post-test, and irregular attendance (up to 3 times absence) in training sessions
Yaghoubitajani et al. 2022 [11]	RCT	UCS	8 Weeks, 3 per week, 50 min	EG: Strengthening CE, CG: Usual daily activity	The program included 5-min warm-up and cool-down, with exercises individualized by demographics, pain (VAS ≤ 5), and progressive overload. Exercises were modified if pain exceeded tolerance. One group trained at home via real-time online supervision, while another trained at the workplace using an exercise diary, with weekly in-person supervision and phone follow-up. The program targeted postural alignment, muscle activation, movement patterns, and scapular stabilization for individuals with upper crossed syndrome and neck–shoulder pain	Neck and shoulder pain and sick leave (VAS), Postural angles (photogrammetry), Muscle activation (EMG, ME-6000 Megawin, 1000 Hz)	(Age: EG: 38.58 ± 7.34, CG: 38.91 ± 3.87), (24 Females and males, EG: 12, CG: 12)	Height: EG: 167 ± 8, CG: 172 ± 9, Weight: EG: 66.33 ± 8.97, CG: 67.18 ± 13.02, BMI: EG: 25.86 ± 1.77, CG: 24.27 ± 2.91	Forward head posture ≥ 45°, forward shoulders ≥ 52°, forward back ≥ 42°) and pain intensity (≥ 3)	Pregnant, had a history of joint diseases in the spine, shoulder, pelvis, and fracture, had surgery during the past year, or had a bodyweight out of the normal range (18 ≥ BMI ≥ 25)
Daneshjoo et al. 2021 [31]	RCT	UCS	9 Weeks, 3 per week	EG: Strengthening CE, CG: Not reported	Corrective games and exercises targeting kyphosis and forward head posture, emphasizing proper neck alignment. Game difficulty was progressively increased by modifying task demands (e.g., players, duration, equipment, distance). Exercises included scapular retraction, chin-tuck–based chest stretching, cervical and thoracic stabilization, spinal mobility, and elastic-resistance back strengthening, with gradual progression of volume and intensity. Duration adapted to individual capacity	Kyphosis angle (flexible ruler), Forward head and shoulder-forward angles (photogrammetry, AutoCAD)	(Age: EG: 13.65 ± 0.74, CG: 14.15 ± 0.87), (40 female students, EG: 20, CG: 20)	Height: EG: 153.05 ± 3.41, CG: 152.70 ± 4.04, Weight: EG: 53.40 ± 2.89, CG: 52.40 ± 4.59, BMI: EG: 22.81 ± 1.30, CG: 22.54 ± 2.60	New York test, head forward of greater than 46 degrees, a kyphosis of greater than 42 degrees, and a shoulder forward of greater than 52 degrees, questionnaires of sports injuries and sports history. Also, the demographic information was gathered by a valid instrument	The subjects did not participate in any other correctional program simultaneously and were within the age range of 13 to 15 years. Also, they were excluded from the investigation in case of any damage or unwillingness to continue working
Darabi et al. 2022 [32]	RCT	UCS	8 Weeks, 3 per week	EG: Strengthening CE, CG: Not reported	Progressive CEs targeting postural alignment, muscle imbalance, and movement control. Exercises included overhead arm elevation holds, shoulder shrugs/rolls, combined cervical–scapular movements, prone trunk and head–chest extension variations, and hanging exercises, with progressive increases in repetitions and duration based on participant tolerance	Kyphosis, forward head, and forward shoulder angles measured (Spine Analysis System, Rasa Pejohan Iran, back surface topography)	(Age: EG: 13.1 ± 1.5, CG: 13.2 ± 2.1), (30 Teenage, EG: 15 boys, CG: 15 boys)	Height: EG: 153.1 ± 1, CG: 153.1 ± 9.6, Weight: EG: 40.9 ± 7.2, CG: 45.9 ± 8.7, BMI: EG: 17.3 ± 2.1, CG: 18.2 ± 7.2	Forward head, forward shoulder, and kyphosis angles greater than 50, 50, and 40 degrees, respectively	History of fracture, surgery, or joint diseases of the spine, pain in the neck or upper limb, and inability to complete the exercise program, history of injury in the last six months, surgery on the lower body, or any complication or defect that leads to disturbance in walking and maintaining balance were also considered as a criterion for exiting the program due to their potential impact on gait analysis
Dehcheshmeh et al. 2022 [34]	RCT	UCS	8 Weeks, 3 per week	EG: Strengthening CE, CG: No intervention	Program including CEs, corrective games, and posture training for upper crossed syndrome. Sessions consisted of a 10-min warm-up, progressively complex exercises, and a 10-min cool-down, with gradual increases in repetitions and duration. Corrective games targeted the activation of weak muscles and the inhibition of overactive muscles, while posture training emphasized head, neck, shoulder, sitting, and standing alignment using verbal, visual, and manual cues. Exercise progression included elastic-ball tasks and stretch exercises with increasing ball size and movement distance over time	Forward head and shoulder angles (photogrammetry, AutoCAD), Kyphosis angle (flexible ruler)	(Age: CG: 26.26 ± 3.43, EG: 25.73 ± 2.23), (30 Males with depression, EG: 15, CG: 15)	Height: EG: 177.93 ± 2.08, CG: 174.00 ± 2.13, Weight: EG:75.33 ± 4.75, CG: 74.60 ± 4.45, BMI: NR	Forward shoulder deformity of more than 52 degrees, a forward head deformity of more than 46 degrees, a kyphosis angle of more than 42 degrees, scoring 17–29 in the Beck questionnaire, male gender	Taking antidepressants while doing exercises, not age range 18 to 40 years, cardiovascular and respiratory diseases, history of surgery in the upper and lower limbs, history of lasting injuries such as degenerative changes in the joints of the body, and presence of upper and lower limbs injuries in previous years
Dehcheshmeh et al. 2024 [33]	RCT	UCS	8 Weeks, 3 per week	EG: Strengthening CE, CG: No intervention	Sahrmann-based protocol with functional and CEs, posture training, and corrective games for UCS. Sessions included 10-min warm-up and cool-down, with exercises progressing simple → complex and gradual load increases. Rest: 45 s between sets, 90 s between exercise types. Program emphasized postural education during daily activities and included deep neck flexor and intrinsic neck stabilization, TheraBand-resisted shoulder exercises, wall-supported abduction/external rotation, and wall slide exercises	Upper extremity function (Y-Balance device), Cervical joint position error (head-mounted device, movements in flexion, extension, lateral rotation, medial rotation)	(Age: EG: 25.73 ± 2.23, CG: 26.26 ± 3.43), (30 Males with depression, EG: 15, CG: 15)	Height: EG: 177.93 ± 2.08, CG: 174.00 ± 2.13, Weight: EG: 75.33 ± 4.75, CG: 74.60 ± 4.45, BMI: NR	Forward shoulder deformity of more than 52 degrees, a forward head deformity of more than 46 degrees, a kyphosis angle of more than 42 degrees, scoring 17–29 in the Beck questionnaire, male gender	Taking antidepressants while doing exercises, not age range 18 to 40 years, cardiovascular and respiratory diseases, history of surgery in the upper and lower limbs, history of lasting injuries such as degenerative changes in the joints of the body, and presence of upper and lower limbs injuries in previous years
Firouzjah et al. 2023 [35]	RCT	Volleyball players with UCS	10 Weeks, 3 per week	EG: Strengthening CE, CG: Regular exercise	Exercises progressed from easy to difficult, with sessions including a 5–10 min neck warm-up, progressive strengthening and stretching (20–60 min), and 5–10 min cool-down. The program targeted kyphosis and forward head posture using muscle release, stretching, and strengthening exercises for the neck, trunk, and shoulders (e.g., SCM/upper trapezius stretching, anterior trunk stretching, Swiss-ball neck and back strengthening, scapular retraction with chin tuck, prone and quadruped stabilization exercises). Exercise intensity progressed by increasing hold duration (10 → 15 s) and TheraBand resistance	Kyphosis angle (flexible ruler), Forward head and shoulder angles (photogrammetry)	(Age: EG: 16.46 ± 0.63, CG: 16.80 ± 0.77), (30 Adolescent volleyball players, EG:15, CG: 15)	Height: EG: 175 ± 3, CG: 175 ± 5, Weight: EG: 74.46 ± 4.10, CG: 71.86 ± 4.79, BMI: EG: 24.19 ± 1.6, CG: 23.37 ± 1.10	All athletes with at least 5 years of regular sports experience in volleyball in the age range of 16–18 years in Babol city, forward head angle of more than 46 degrees, forward shoulder angle of more than 52 degrees, and kyphosis of more than 40 degrees, with no special injury	Injury during the exercise or training period, absence in at least 3 sessions of training, history of back pain, or any special injury
Hesar et al. 2023 [36]	RCT	UCS	8 Weeks, 3 per week, 30–60 min	EG: Strengthening CE, CG: Not reported	CE intervention—Janda’s muscle chain-based CE program with progressive overload; Protocol: 8-week program, 3 sessions per week, each session 30–60 min, supervised in a clinical setting, preceded by a 1-week familiarization phase; exercises individually tailored based on postural deviation patterns (pelvis, lumbar, thoracic, cervical regions), gradual increase of holding time (6 to 30 s) and repetitions (6 to 12 per set), with continuous feedback, 5-min warm-up and cool-down each session; Tool/Brand: not specified (bodyweight CEs, no equipment reported)	Shoulder proprioception (ROM, flexion, abduction, internal/external rotation, Leighton Flexo meter), Shoulder active ROM (sitting, trunk stabilized, Leighton Flexo meter), Upper extremity function (Y-Balance Test Upper Quarter), Forward head posture (goniometer), forward shoulder posture (ruler distance from wall), Thoracic kyphosis (flexible ruler)	(Age: EG: 16 ± 2.1, CG:16 ± 3.2), (30 Females student, E,C:15)	Height: EG: 169.3 ± 4.1, CG: 166.2 ± 32, Weight: EG: 64.4 ± 2.3, CG: 63.2 ± 1.2, BMI: EG: 23.34 ± 14.4, CG: 22.24 ± 14.34	Female students aged 11 to 14 years in Urmia, possessing general health, presence of upper crossed syndrome abnormalities with criteria of forward head angles, forward shoulders, and kyphosis greater than 62, 46, and 42 degrees, respectively, no history of dislocation or fracture in the upper limbs in the past year, no history of any surgery in the upper limb area, no history of neurological or musculoskeletal diseases that limit movement, and no pain in the upper limbs before or during the tests	Consumption of sedatives and estrogen-containing medications, alcohol intake, experiencing severe pain in the upper limbs during tests, contracting an illness or injury that prevents participation in the program
Ahmed et al. 2022 [50]	RCT	UCS	8 Weeks, 3 per week	EG: Strengthening CE, and myofascial release, CG: Myofascial release only	Group 1: on the anterior chest (pectoralis major and minor, levator scapulae, and sternocleidomastoid), and the posterior part (upper trapezius). For releasing, cross hand technique was applied with mild pressure of about 90 s-120 s. Accordingly, MFR therapy was applied two times, each time for 90 s on the determined areas. With seven minutes of hot pack at the upper back and anterior chest, and patient education about faulty posture was used as baseline treatment. Group B performed CEs plus MFR therapy under the direct supervision of the therapist. For the CE plus MFR group, the training protocol included four parts: warming up, MFR therapy, main exercises, and baseline treatment	Forward head and forward shoulder angles (photography, Sony 16.1 MP digital camera), Function (Neck Disability Index), Pain (VAS)	(Age: EG: 24.17 ± 3.05, CG:24.75 ± 3.05), (30 Females, EG:15, CG:15)	Height: NA, Weight: EG: 71.92 ± 11.13; CG: 68.67 ± 8.195, BMI: EG: 24.58 ± 2.14; CG: 24.62 ± 1.723	Forward head posture angle greater than 45°, a forward shoulder distance greater than 5.6 cm, mild to moderate pain (VAS score 10–60 mm), and willingness to participate in the trial	History of disease, fractures, surgeries, joint issues, spinal injuries, abnormal BMI, diagnosed muscular pathology, cervical soft tissue fractures, or those unable to attend at least three exercise sessions per week
Karimian et al. 2019 [37]	RCT	UCS	12 Weeks, 3 per week, 45–60 min	EG: NASM CE, CG: Usual daily activity	The program combined self-myofascial release, stretching, strengthening, and dynamic exercises targeting the latissimus dorsi, thoracic spine, upper trapezius, SCM, and levator scapulae. Stretching focused on SCM, levator scapulae, upper trapezius, pectorals, and latissimus dorsi to the pain threshold. Strengthening emphasizes deep cervical flexors, scapular stabilizers, and the posterior chain (e.g., chin tucks, prone scaption, quadruped, and ball exercises) to fatigue. Dynamic exercises incorporated cervical retraction with functional movements and compound lifts. Exercise intensity, repetitions, and duration progressed gradually. Ergonomic training included an educational pamphlet to reduce musculoskeletal load during manual tasks	Forward head, forward shoulder, and kyphosis angles (photogrammetry)	(Age: EG: 45.2 ± 8.1, CG: 44.1 ± 7.8), (23 Non-athletic teachers EG: 12, CG: 11)	Height: EG: 178.8 ± 6.5, GC: 175.8 ± 7.0, Weight: EG: 78.3 ± 13, CG: 77.9 ± 13.0, BMI: NR	Hyper-kyphosis of more than 42 degrees, forward head posture of more than 45 degrees, and forward shoulder posture of more than 52 degrees	Pathologic symptoms such as a history of surgery, fracture or joint diseases of the spine, osteoporosis, acute rheumatoid arthritis, blood diseases, congestive heart failure, malignancy, severe skin sensitization, and athletic activities
Khazaei et al. 2022 [38]	RCT	UCS	8 Weeks, 3 per week, 40–60 min	EG: Strengthening CE, CG: Not reported	Corrective and physio ball exercise intervention-Protocol: 8-week supervised program, 3 sessions per week; each session duration 40–60 min including 5–10 min warm-up, 10–15 min stretching of shortened muscles (especially anterior chest and shoulder muscles) with 12–20 s hold per repetition to tolerable stretch, followed by 20–30 min corrective and strengthening exercises (based on corrective movement principles) and 5 min cool-down; exercise volume (repetitions and holding time) and rest intervals between sets were progressively increased across the 8 weeks according to individual characteristics and tolerance; all exercises were performed under direct examiner supervision; Tool/Brand: physio ball (Swiss ball, brand not reported), bodyweight CE equipment	Surface EMG of rhomboid major, middle trapezius, serratus anterior (ME6000 system)	(Age: EG: 16.93 ± 0.88, CG: 17.00 ± 0.75), (30 Males, EG: 15, CG: 15)	Height: EG: 163.93 ± 2.01, CG:161.66 ± 6.85, Weight: EG: 63.40 ± 10.09, CE: 65.20 ± 6.51, BMI: EG: 25.10 ± 2.76, CG: 27.88 ± 3.84	Forward head, forward shoulder	History of mental illnesses, fractures, surgeries, or joint diseases of the spine, or any pain in the neck or upper limbs, fractures, and neuromuscular problems during the study, and missing two sessions
Khosrojerdi et al. 2022 [39]	RCT	UCS	8 Weeks, 3 per week, 30–60 min	EG: Strengthening CE, CG: Monthly health education	Exercise protocol, 8-week intervention, 3 sessions per week; each session 30–60 min; structure: 5–10 min warm-up, followed by stretching of shortened muscles (pectoralis major/minor, sternocleidomastoid, upper deltoid, intercostals, upper cervical extensors) and strengthening of weakened muscles (scapular protractors, deep cervical flexors, lower cervical extensors, thoracic spine extensors); exercises performed repetitively with progressive overload across weeks; postural re-education integrated through 1–2 instructional sessions plus daily practice reminders; combined protocol included supervised CEs plus daily postural habit training reinforced by short videos (20–30 s) viewed once daily and posture practice ≥ 3 times/day; no external resistance equipment specified (bodyweight-based CEs)	Thoracic kyphosis (flexible ruler), Forward head and forward shoulder angle (photogrammetry)	Age: not reported, 30 Female employees, EG: 15, CG: 15	Height: NR, Weight: NR, BMI: NR	Postural abnormalities of kyphosis, more 46/83, head forward 46, and shoulder forward posture 52 (simultaneously), and willingness to participate	Observation of any pathological symptoms, history of fracture, surgery, joint disease, injuries to the cervical and dorsal spine and shoulder girdle, musculoskeletal disorders, lower extremity cross syndrome, abnormal BMI, and regular physical activity for at least 6 h per week resulted in withdrawal
Maarouf et al. 2020 [40]	RCT	Wheelchair basketball players with UCS	8 Weeks, 5 per week, 60 min	EG: Strengthening CE, CG: Regular exercise	Exercise protocol, Each session up to 60 min including 10 min warm-up, 10 min stretching and range-of-motion exercises, up to 35 min progressive resistance/strength training, 15 min cool-down, and postural exercises focusing on scapular stabilization; all exercises demonstrated and technique fully instructed, reinforced with illustrated educational booklet specifying movements and repetitions; program based on Harborview Medical Center progressive resistance exercises for individuals with spinal cord injury and ACSM exercise prescription guidelines; sessions supervised by a certified trainer; Tools/Brands: Gym equipment (unspecified), instructional booklet with images	Forward shoulder and forward head angles (Photography, AutoCAD), thoracic kyphosis (Flexible ruler)	(Age: EG: 39.08 ± 5.08, CG: 43 ± 41.23), (24 Wheelchair basketball athletes, E: 12, CG: 12)	Height: EG: 77.25 ± 3.22, CG: 73.95 ± 8.24, Weight: EG: 54.5 ± 8.73, CG: 69.91 ± 1.24, BMI: NR	Thoracic kyphosis exceeding 42 degrees, forward head posture greater than 45 degrees, and forward shoulders exceeding 52 degrees, without pain	NA
Mansouri et al. 2023 [10]	RCT	UCS	8 Weeks, 3 per week, 20 min	EG: Strengthening CE, CG: No intervention	Consisted of three 20-min sessions combining stretching and isometric exercises targeting the anterior and posterior neck extensors and deep neck flexors, aimed at improving cervical-TMJ joint function and correcting upper quadrant muscle imbalances and misalignment, performed at low intensity tailored for elderly participants and following ACSM guidelines	Pain (Numerical rating scale, NRS at baseline), Cervical extension (occiput-to-wall distance, measured with tape)	30 Elderly women, EG: 15, CG: 15	Height: NR, Weight: NR, BMI: NR	TMJ Pain, participating volunteers	NA
Manzari et al. 2021 (1) [41]	RCT	UCS	8 Weeks, 3 per week, 30–70 min	EG: Strengthening CE, CG: No intervention	Included shoulder girdles (levator scapulae, trapezius, SCM, pectorals, latissimus dorsi, cervical/scapular muscles) and pelvic girdles (deep and transverse abdominals, multifidus, internal obliques) programs, plus a combined group addressing the whole spine. Programs lasted 8 weeks, three 30–70 min sessions per week, progressing in intensity, repetitions, and duration. Stretching to pain threshold; strengthening to exhaustion, with gradual load increase under supervision	Forward head and shoulder angles (Photography, AutoCAD 2010), Thoracic kyphosis (Flex curve ruler)	(Age: EG: 19.53 ± 1.12, CG: 19.53 ± 1.12), (30 Females students, EG: 15, CG:15)	Height: EG: 167.07 ± 5.88, CG: 167.07 ± 5.88, Weight: EG: 60.53 ± 7.59, CG: 60.53 ± 7.59, BMI: NR	Forward head posture ≥ 46°, forward shoulder posture ≥ 52° and hyper-kyphosis ≥ 42°	Neurological disease, scoliosis, failing to attend pre- and post-test, and irregular attendance (up to 3 times) in training sessions
Manzari et al. 2021 (2) [42]	RCT	UCS	8 Weeks, 3 per week, 30–70 min	EG: Strengthening CE, CG: No intervention	CE program, Included two types: localized corrective (targeting either shoulder girdle or lumbar girdle) and comprehensive corrective (combined shoulder and lumbar girdle); Protocol: 8-week program, 3 sessions per week, 30–70 min per session, supervised by an examiner; exercises progressed from simple to complex with gradual increases in intensity, repetitions, duration, and load; shoulder girdle program focused on stretching shortened muscles and strengthening weak muscles using tools such as stick, resistance band, cable, weights, foam roller, and Swiss ball; lumbar girdle program focused on core stability for improved biomechanics, force generation, and balance using gradual activation of deep trunk muscles and tools like stick, weights, and Swiss ball for balance; combined program integrated both shoulder and lumbar programs simultaneously; all sessions included warm-up, CEs, and cool-down; exercise volume and duration standardized across all groups to ensure comparability	Forward head and forward shoulder (Photogrammetry, AutoCAD), Thoracic kyphosis (flexible ruler), Static balance (RS scan International Footscan7 pressure plate, 300 Hz, COP)	(Age: EG:19.53 ± 1.12, CG: 19.53 ± 1.12), (30 Female students, EG: 15, CG:15)	Height: EG: 167.07 ± 5.88, CG: 167.07 ± 5.88, Weight: EG: 60.53 ± 7.59, CE: 60.53 ± 7.59, BMI: NR	Forward head posture ≥ 46°, forward shoulder posture ≥ 52° and hyper-kyphosis ≥ 42°	Neurological disease, scoliosis, failing to attend pre- and post-test, and irregular attendance (up to 3 times) in training sessions
Seyedahmadi et al. 2025 [49]	RCT	UCS	8 Weeks, 3 per week	EG: Strengthening CE, CG: No intervention	The 8-week land-based CE program for women with UCS included three supervised 40–60 min sessions per week with 5–10 min warm-up, 30–45 min CEs, and 5–10 min cool-down, combining stretching of tight muscles, strengthening of weak muscles, mobility training with dynamic movements and progressively larger foam rollers, and proprioception/postural control, with gradual increases in repetitions, isometric hold duration, and resistance tailored to individual needs, while the 8-week water-based program included three weekly sessions with 10–15 min warm-up, 35–45 min exercises, and 5–10 min cool-down, progressing through environmental normalization with water massage and myofascial release, muscle balance restoration with stretching, corrective breathing, strengthening, and proprioception exercises, and functional integration through water-based ball games emphasizing correct posture, all performed under specialist supervision	Thoracic kyphosis (Flexible ruler), forward shoulder, Forward head angles (Photography)	(Age: EG: 45.2 ± 8.1, CG: 44.1 ± 7.8), (23 non-athletic teachers EG: 12, CG: 11)	Height: EG: 178.8 ± 6.5, CG: 175.8 ± 7.0, Weight: EG: 78.3 ± 13, CG: 77.9 ± 13.0, BMI: NR	Shoulder angle (SA > 49°); cervical angle (CA > 44°), thoracic kyphosis angle (TKA > 42°), 35; activity history between 2 to 5 years; normal body mass index, no other abnormalities (except upper crossed syndrome)	Having significant neurologic or cardiovascular disorders; (2) a history of surgery on the upper limbs in the previous six months; (3) the beginning of any analgesic intervention for musculoskeletal pain within the previous six weeks
Sakinepoor et al. 2024 [43]	RCT	Handball players with UCS	8 Weeks, 3 per week, 60 min	EG: Strengthening CE, CG: Regular exercise	CE program, 8-week intervention, 3 sessions per week, 1 h per session (10 min warm-up, 40 min main exercises, 10 min cool-down); program applied to experimental group, control group received no treatment; protocol based on previous studies, combining two prior exercise approaches; resistance exercises started with low load, 2 sets of 10 repetitions in weeks 1–2, progressed gradually to 3 sets of 12 reps in weeks 3–5, and 3 sets of 15 reps in weeks 6–8, with 30 s rest between sets; stretching exercises held initially for 13 s, increased by 2 s each week, sets of stretches progressed similarly, 30 s rest between sets; exercises selected and progressed to integrate strengthening and stretching from two prior study protocols	Muscle activity (EMG, Myon wireless system, Ag/Cl electrodes, F-RG Germany)	(Age: EG: 17.60 ± 1.80, CG: 16.66 ± 1.87), (30 Male handball players, EG: 15, CG: 15)	Height: EG: 173.06 ± 4.31, CG: 171.46 ± 5.38, Weight: EG: 70.13 ± 3.97, CG: 69.80 ± 4.94, BMI: EG: 23.07 ± 1.10, CG: 23.65 ± 0.36	Forward head posture exceeding 46 degrees, forward shoulders greater than 52 degrees, kyphosis greater than 42 degrees, age range of 14 to 20 years, normal body mass index, male gender, and a history of 5 years of handball activity	Pathological symptoms related to a history of fractures, surgeries, or spinal joint diseases, having any impactful abnormalities other than upper crossed syndrome, experiencing any pain in the neck or upper limbs, failure to complete the exercise program as per the study, or lack of interest from subjects to continue participating in the study
Mokhtaran et al. 2025 [47]	RCT	UCS	6 Weeks	EG: NASM CE, CG: Usual daily activity	Eccentric-based exercise protocol involved controlled muscle lengthening through functional movements, with gradual progression by increasing resistance and extending the eccentric phase from 3 to 5 s per repetition, aligned with NASM training phases and supervised by 2–3 biomechanical experts	Thoracic kyphosis angle (kyphometer), Forward head angle and shoulder angle (Photogrammetry)	(Age: EG: 13.92 ± 0.9, CG: 15.71 ± 0.72), (Females, EG a: 12, EG b: 14, CG: 14)	Height: EG a: 162.25 ± 8.97, EG b: 158.64 ± 5.32, CG: 164.86 ± 4.88, Weight: EG a: 51.33 ± 9.80, EG b: 50.00 ± 11.8, CG: 62.00 ± 8.89, BMI: EG a: 19.36 ± 2.77, EG b: 19.84 ± 4.46, CG: 20.84 ± 3.42	Female middle school students with thoracic kyphosis > 32°	A history of spinal fractures or surgeries, psychological issues, structural kyphosis, neuromuscular diseases, abnormalities in the spine, or physical disorders were not allowed to participate in the study. A history of chronic pain in the cervical and lumbopelvic regions, a history of postural control disorders, participation in professional or regular sports, severe visual impairments, and any medical condition that would prevent participation in physical training programs, if they missed practice for three nonconsecutive sessions or two consecutive sessions
Salamat et al. 2020 [48]	RCT	UCS	8 Weeks, 3 per week	EG: Strengthening CE, CG: Usual daily activity	The CE and functional play program lasted 8 weeks, with three 60-min sessions per week. The control group only performed daily activities. The program targeted all three abnormalities of upper crossed syndrome simultaneously, focusing on activating weak muscles and inhibiting overactive muscles. The corrective games were custom-designed, scientifically forward, age-appropriate, and approved by experts in CE and sports medicine	Thoracic kyphosis (flexible ruler), Forward head and forward shoulder angles (Photography)	(Age: EG: 11.50 ± 1.16, CG: 11.66 ± 1.07), (24 Male students, EG: 12, CG: 12)	Height: EG: 147 ± 7.54, CG: 148.5 ± 6.48, Weight: EG: 38.67 ± 6.97, CG: 40.08 ± 5.64, BMI: NR	Male students aged 10 to 13 years, with three abnormalities: forward head posture with an angle equal to or greater than 46 degrees, kyphosis with an angle greater than 40 degrees, and forward shoulders with an angle equal to or greater than 52 degrees. Additionally, they have no history of fractures, no history of surgeries, no spinal joint diseases, no lumbar hyper-lordosis, no cardiovascular or respiratory disorders, and no pathological pain in the neck or upper limbs	Absence of samples for more than three sessions in the training program
Shadi et al. 2024 [44]	RCT	UCS	6 Weeks, 3 per week, 60 min	EG: Strengthening CE, CG: No intervention	The CE exercises were done in the gym, and the corrective movements specialist was responsible for supervising their performance. Selected CE was designed to correct posture and address the mentioned abnormalities through stretching exercises for shortened muscles and strengthening exercises for individuals with weak muscles. These exercises included a 5–10-min warm-up followed by stretching exercises for the chest, hip flexor-psoas, upper trapezius, intercostal muscles, upper neck extensors, and then strengthening exercises for the shoulder protractors, deep neck flexors, lower neck extensors, and thoracic spine extensors	Thoracic kyphosis (Flex curve ruler), forward shoulder and forward head angles (Photography)	(Age: EG: 14.66 ± 0.72, CG: 14.66 ± 0.72(, (30 Female students, EG: 15, CG:15)	EG and CG: Height: 7.79 ± 151.46, Weight: 7.69 ± 45.40, BMI: NR	Simultaneously, they have postural abnormalities such as kyphosis, FH, and RS, and express a willingness to participate	Signs of illness, fractures, surgeries, joint problems, injuries in the spine, skeletal-muscular imbalances, lower limb cross syndrome, abnormal BMI, or engaging in regular physical activity for at least 6 h per week
Shahrjerdi et al. 2024 [45]	RCT	UCS	8 Weeks	EG: Strengthening CE, CG: Usual daily activity	The experimental group underwent an 8-week selective CE program for UCS, with three 50-min supervised sessions per week, including standard warm-up and cool-down. Exercises combined stretching, strengthening, and stabilization targeting forward head, forward shoulders, and kyphosis, following established UCS protocols and ACSM guidelines. The program progressed through three phases: an initial two-week phase with 7–10 sets of 10–15 s holds, an improvement phase with 5–6 sets of 10–15 repetitions, and a final two-week maintenance phase at consistent intensity, all performed under trained instructor supervision. The control group received no CEs and continued their usual daily activities, with all participants completing a work productivity questionnaire	Secondary outcomes (Forward head and forward shoulder angles measured with goniometer, Thoracic kyphosis measured with flexible ruler)	(Age: EG: 36.86 ± 5.88, CG: 37.19 ± 7.94), (25 Males, 23 females, EG: 23, CG: 25)	Height: NR, Weight: EG: 77.86 ± 15.94, CG: 77.31 ± 17.11, BMI: EG: 25.85 ± 3.51, CG: 25.95 ± 3.61	Not having any injuries that could hinder their participation in an exercise program, having the capability to engage in the stretching, strengthening, and stabilization exercises, having a minimum of six months of working experience, and being workers (such as electrical linemen, technicians, and other work-related activities) with a minimum of 6 and a maximum of 12 h of work per day	More than three sessions or a non-agreement to continue with the research, the presence of acute musculoskeletal disorders or other illnesses, and pregnancy in female participants
Rezaei et al. 2025 [46]	RCT	Swimmers with UCS	8 Weeks, 3 per week, 30–40 min	EG: Strengthening CE, CG: Regular exercise	The experimental group performed an 8-week swimmer-specific CE program with three 30–40 min sessions per week, conducted in the gym before regular training under the supervision of a physical therapist. Exercises emphasized flexibility and muscle strengthening, including chin tuck, latissimus dorsi, scalene, and pectoral stretches, scapular-squeeze, trunk rotation, external rotation, scapular retraction, Y-raise, dynamic latissimus dorsi and W stretch, push-up with trunk rotation, pectoral dynamic stretch, superman, pelvic tilt, wall push-up, bird dog, head lift with neck curl, sleeper stretch, and levator scapulae and trapezius stretch. Intensity progressed over eight weeks by increasing repetitions, duration, and number of movements. The control group continued regular swimming training without CEs	Forward head and forward shoulder angles (Photogrammetry, AutoCAD 2018), Thoracic kyphosis (flexible ruler), Shoulder pain (VAS), Active shoulder ROM (flexion, extension, internal/external rotation, universal goniometer)	(Age: EG: 28.85 ± 6.49, CG: 27.66 ± 6.19), (30 Male professional swimmers)	Height: NR, Weight: EG: 80.07, CG:81.60, BMI: NR	Forward head angle > 45°, forward shoulder angle > 52°; kyphosis > 40°; self-reported shoulder pain (VAS 1–7); minimum of 3 regular training sessions per week; no history of spinal or upper limb surgery; and at least 3 years of front crawl swimming experience	Any serious injury during the study, absence from post-test assessment, or missing three consecutive training sessions during the intervention

*UCS* Upper Crossed Syndrome, *NR* Not Reported, *EG* Experimental Group, *CG* Control Group, *CE* Corrective Exercise, *RCT* Randomized Controlled Trials, *VAS* Visual Analogue Scale, *EMG* Electromyograp

Across the included studies, postural variables were assessed using largely consistent and standardized protocols. Thoracic kyphosis was predominantly measured with a flexible ruler (flexicurve method), typically molded along the spinous processes from T2 to T12 in relaxed standing, traced onto paper, and calculated using the formula θ = 4 arctan(2H/L); this approach was the most frequently used method, whereas photogrammetric analysis and kyphometer were used less commonly. Forward head and forward shoulder angle were assessed mainly through lateral digital photogrammetry, representing the most standardized measurement approach across studies; reflective markers were commonly placed on the tragus, C7, and acromion, participants stood (or occasionally sat) in a relaxed posture at a fixed distance from the wall and camera, three images were typically captured, and angles (craniovertebral angle for FHP and C7-acromion angle for forward shoulder angle) were calculated using software such as AutoCAD or Kinovea. Pain was primarily evaluated using the VAS, with only a few studies employing the Numeric Rating Scale. Static balance was assessed using instrumented systems such as the Biodex Balance System or force/pressure platforms to quantify center-of-pressure displacement under standardized conditions (e.g., eyes open/closed, single-leg stance), while dynamic balance was most frequently measured using the Y-Balance Test with normalized reach distances. Muscle activity was evaluated in a limited number of studies using sEMG following SENIAM guidelines, commonly normalized to MVIC. Functional outcomes and disability were assessed using heterogeneous questionnaires (e.g., neck-, shoulder-, or general health-specific scales) without a single dominant instrument. Overall, flexible ruler assessment for kyphosis and lateral photogrammetry for forward head and forward shoulder angles were clearly the most prevalent and methodologically consistent tools across the included studies.

### Quality assessment

Table 3 shows the results of the quality assessment using the PEDro scale, ranging from 5/10 to 8/10. The highest studies, Sakinepoor et al. 2024 [43] and Shahrjerdi et al. 2024 [45], achieved a good rating (PEDro = 8/10). The lowest-rated studies were Hesar et al. 2023 [36], and Khosrojerdi et al. 2022 [39]; the overall quality was fair (PEDro = 6.14/10). The methodological quality and risk of bias of included studies were independently assessed using the RoB-2 scale [15] for randomized trials in Table 4 [49]. The low-risk studies were Yaghoubitajani et al. 2022 [11], Mokhtaran et al. 2025 [47], Shahrjerdi et al. 2024 [45], Rezaei et al. 2025 [46], and high-risk studies were Hesar et al. 2023 [36], Khosrojerdi et al. 2022 [39]. The following domains received lower overall risk: randomization process, missing outcome data, selection of the reported result, and higher risks were in deviations from the intended intervention and measurement of the outcome. GRADE assessment showed moderate-certainty evidence for improvements in postural alignment outcomes (forward head angle, forward shoulder angle, and thoracic kyphosis). High-certainty evidence supported improvements in static balance, while low-certainty evidence was found for dynamic balance. The certainty of evidence for muscle activation outcomes ranged from moderate to very low, largely due to inconsistency and imprecision. Very low-certainty evidence was identified for pain outcomes, indicating substantial uncertainty regarding the effects of CEs on pain (Supplementary Material 1).

**Table 3:** Quality assessment of included studies using the PEDro scale

Authors	Random allocation	Concealed allocation	Baseline comparability	Blinding of participants	Blinding of therapists	Blinding of assessors	Adequate of follow-up (> 85%)	Intention-to-treat analysis	Between-group statistical comparisons	Reporting point measures and measures of variability	Overall
Taghi Aghaei et al. 2024 [13]	1	0	1	0	0	0	1	1	1	1	6
Abdolahzadeh et al. 2019 [27]	1	0	1	0	0	0	1	1	1	1	6
Ahmadi et al. 2019 [30]	1	0	1	0	0	0	1	1	1	1	6
Ahmadi et al. 2020 [28]	1	0	1	0	0	0	1	1	1	1	6
Ahmadi et al. 2022 [29]	1	0	1	0	0	0	1	1	1	1	6
Arshadi et al. 2019 [12]	1	0	1	0	0	0	1	1	1	1	6
Yaghoubitajani et al. 2022 [11]	1	0	1	0	0	0	1	1	1	1	6
Daneshjoo et al. 2021 [31]	1	0	1	0	0	0	1	1	1	1	6
Darabi et al. 2022 [32]	1	0	1	0	0	0	1	1	1	1	6
Dehcheshmeh et al. 2022 [34]	1	0	1	0	0	0	1	1	1	1	6
Dehcheshmeh et al. 2024 [33]	1	0	1	0	0	0	1	1	1	1	6
Firouzjah et al. 2023 [35]	1	0	1	0	0	0	1	1	1	1	6
Hesar et al. 2023 [36]	0	0	1	0	0	0	1	1	1	1	5
Ahmed et al. 2022 [50]	1	0	1	1	0	0	1	1	1	1	7
Karimian et al. 2019 [37]	1	0	1	0	0	0	1	1	1	1	6
Khazaei et al. 2022 [38]	1	0	1	0	0	0	1	1	1	1	6
Khosrojerdi et al. 2022 [39]	0	0	1	0	0	0	1	1	1	1	5
Maarouf et al. 2020 [40]	1	0	1	0	0	0	1	1	1	1	6
Mansouri et al. 2023 [10]	1	0	1	0	0	0	1	1	1	1	6
Manzari et al. 2021 (1) [41]	1	0	1	0	0	0	1	1	1	1	6
Manzari et al. 2021 (2) [42]	1	0	1	0	0	0	1	1	1	1	6
Sakinepoor et al. 2024 [43]	1	0	1	1	1	0	1	1	1	1	8
Seyedahmadi et al. 2025 [49]	1	0	1	1	0	0	1	1	1	1	7
Rezaei et al. 2025 [46]	1	0	1	0	0	0	1	1	1	1	6
Salamat et al. 2020 [48]	1	0	1	0	0	0	1	1	1	1	6
Shadi et al. 2024 [44]	1	0	1	0	0	0	1	1	1	1	6
Shahrjerdi et al. 2024 [45]	1	0	1	1	1	0	1	1	1	1	8
Mokhtaran et al. 2025 [47]	1	0	1	0	0	0	1	1	1	1	6
Overall											6.14

**Table 4:**
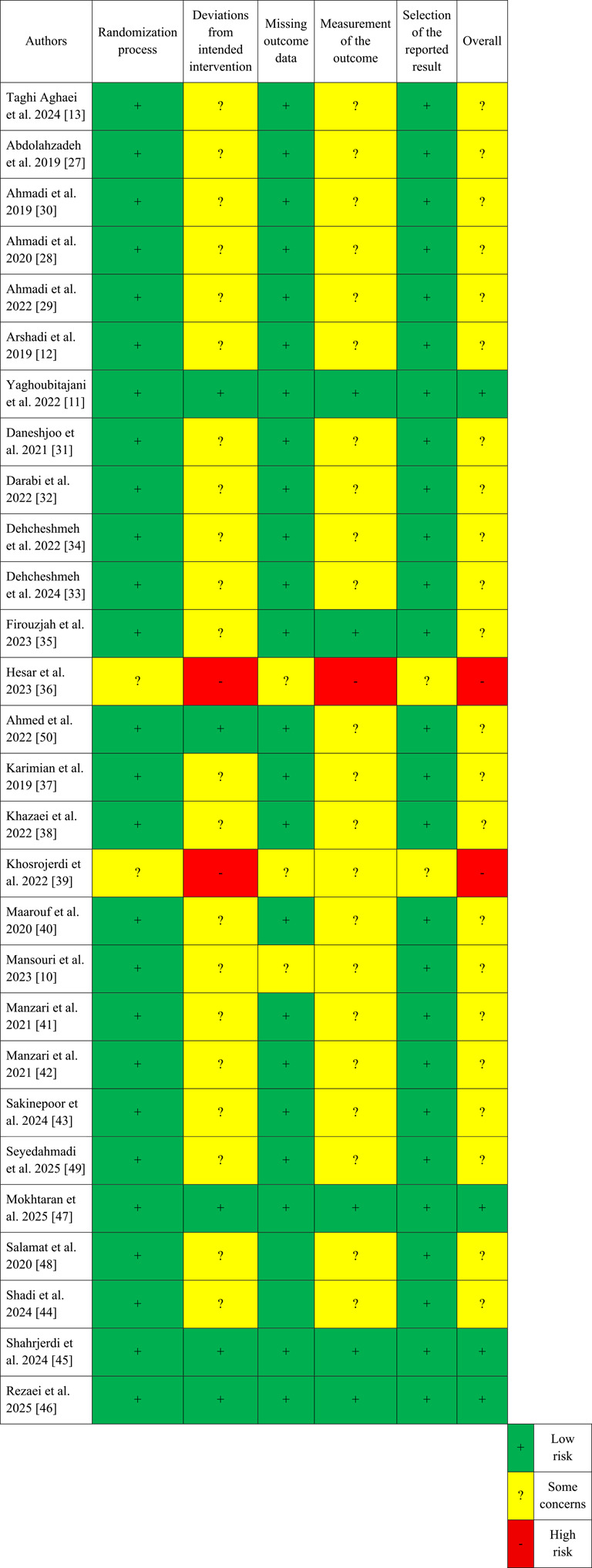
Risk of bias for included studies using the RoB-2 scale [10–13, 27–49]

### Outcome measured

Out of the 28 studies [10–13, 27–50], 18 measured forward head [11, 13, 27, 29, 31, 32, 34, 35, 37, 39, 40, 43–46, 48–50], 18 targeted forward shoulder [11–13, 27, 29, 31, 32, 34, 35, 37, 39, 40, 43–46, 49, 50], 18 targeted hyperkyphosis [11–13, 27, 29, 31, 32, 34, 35, 37, 39, 40, 43–47, 49], 3 assessed muscle activation [11, 12, 38], and 4 reported function [10, 30, 33, 36], 4 targeted pain [10, 11, 46, 50], and 3 studies measured balance [13, 28, 42].

### Posture

**Fig. 2:**
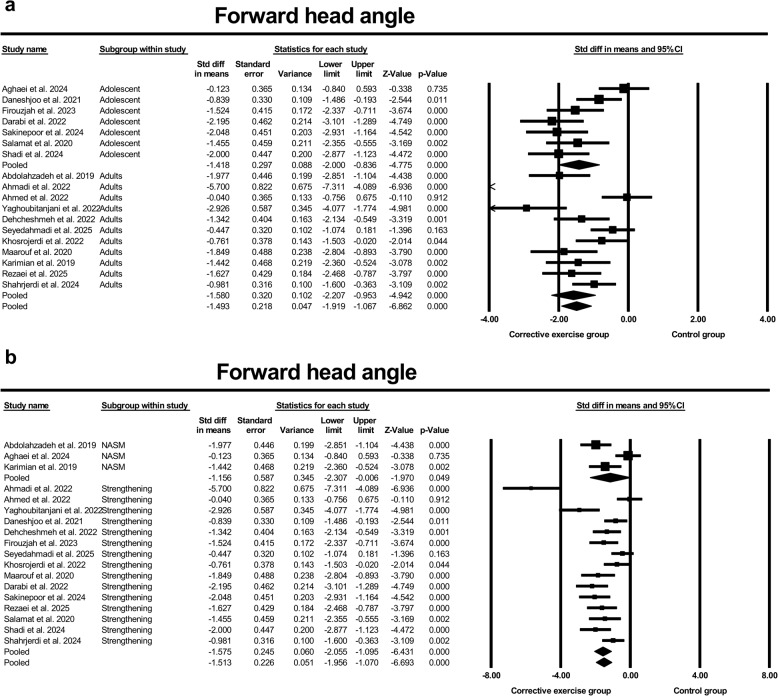
**a** Meta-analysis results of the forward head angle comparing the corrective exercise and the control groups, including age subgroup analysis. **b** Meta-analysis results of the forward head angle comparing the corrective exercise and the control groups, including exercise type subgroup analysis

Eighteen studies investigated the effect of the CE on the forward head, by the Craniovertebral (°) (CVA) [11, 13, 27, 29, 31, 32, 34, 35, 37, 39, 40, 43–46, 48–50]. The results of the meta-analysis suggested strong evidence of significant decreases in forward head angle in combined adolescent and adults (SMD: −1.49; 95% CI: −1.91 to −1.06; *p* < 0.01) with high heterogeneity (I^2^ = 79%, *p* < 0.01), adolescent (SMD: −1.41; 95% CI: −2.00 to −0.83; *p* < 0.01) with high heterogeneity (I^2^ = 72%, *p* < 0.01), and adults (SMD: −1.58; 95% CI: −2.20 to −0.95; *p* < 0.01) with high heterogeneity (I^2^ = 83%, *p* < 0.01) (Fig. 2a) in the CE group compared to the control group.

Subgroup analysis for the effect of exercise type showed that although NASM and strengthening exercise both are both effective in reducing forward head angles. However, strengthening exercise retrieved larger effect size (SMD: −1.57; 95% CI: −2.05 to −1.09; *p* < 0.05) with high heterogeneity (I^2^ = 77%, *p* < 0.01) compared to NASM (SMD: −1.16; 95% CI: −2.31 to −0.006; *p* < 0.05) with high heterogeneity (I^2^ = 82%, *p* < 0.01) (Fig. 2b). These findings warrant cautious interpretation due to the small number of studies assessing NASM CE.

To examine whether the training environment influences the effects of CE, we compared studies implementing aquatic versus land-based protocols. Only Ahmadi et al. 2022, which reported the highest effect size (SMD: − 5.70; 95% CI: − 7.31 to − 4.08; *p* < 0.01), conducted CE as aquatic training; however, all other studies employed land-based training.

Furthermore, no evidence of publication bias was detected based on the funnel plot and Egger’s test, although the limited number of included studies may reduce the sensitivity of these assessments. Meta-regression analysis did not identify a significant moderating effect of intervention duration (6 to 12 weeks) on the pooled effect size (95% CI: −4.73 to 1.75; *p* = 0.33).

**Fig. 3:**
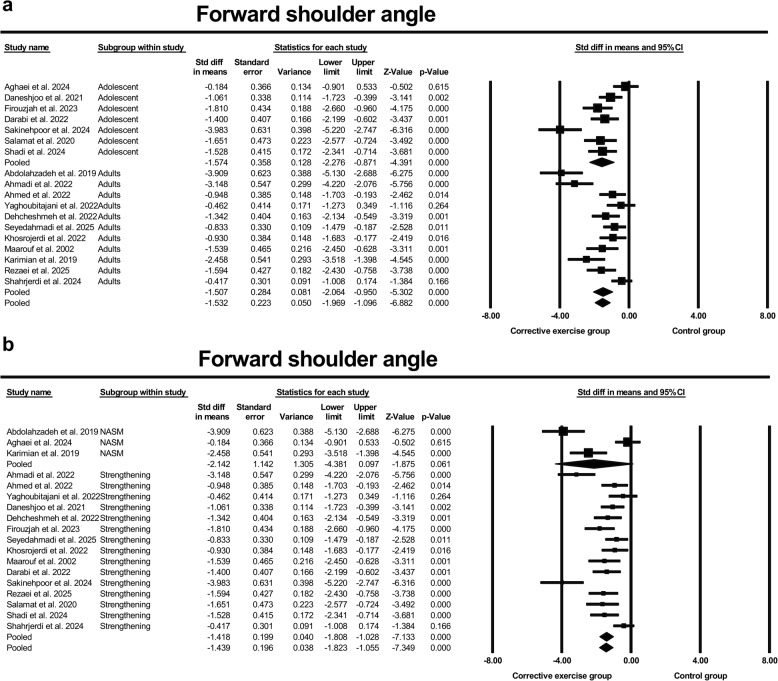
**a** Meta-analysis results of the forward shoulder angle comparing the corrective exercise and the control groups, including age subgroup analysis. **b** Meta-analysis results of the forward shoulder angle comparing the corrective exercise and the control groups, including exercise type subgroup analysis

Eighteen studies investigated the effect of the CE on forward shoulder (°), using photography and photogrammetry [11–13, 27, 29, 31, 32, 34, 35, 37, 39, 40, 43–46, 49, 50]. The results of the meta-analysis suggested strong evidence of significant decreases in forward shoulder angle in combined adolescent and adults (SMD: −1.53; 95% CI: −1.96 to −1.09; *p* < 0.01) with high heterogeneity (I^2^ = 78%, *p* < 0.01), adolescent (SMD: −1.57; 95% CI: −2.27 to −0.87; *p* < 0.01) with high heterogeneity (I^2^ = 80%, *p* < 0.01), and adults (SMD: −1.50; 95% CI: −2.06 to −0.95; *p* < 0.01) with high heterogeneity (I^2^ = 80%, *p* < 0.01) (Fig. 3a) in the CE group compared to the control group.

Subgroup analysis for the effect of exercise type showed that strengthening exercise suggested a significant difference in reducing forward shoulder angle in the CE compared to the control group (SMD: −1.41; 95% CI: −1.80 to −1.02; *p* < 0.05) with high heterogeneity (I^2^ = 73%, *p* < 0.01). However, subgroup analysis indicated no meaningful difference in NASM CE (SMD: −2.14; 95% CI: −4.38 to 0.10; *p* = 0.06) with high heterogeneity (I^2^ = 93%, *p* < 0.01) (Fig. 3b). These findings warrant cautious interpretation due to the small number of studies assessing NASM CE.

To compare the effects of aquatic- and land-based CE on forward shoulder angle, only Ahmadi et al. 2022 (SMD: −3.14; 95% CI: −4.22 to −2.07; *p* < 0.01) implemented aquatic-based CE. In contrast, Sakinehpoor et al. 2024 (SMD: −3.98; 95% CI: −5.22 to −2.74; *p* < 0.01) and Abdolahzadeh et al. 2019 (SMD: −3.90; 95% CI: −5.13 to −2.68; *p* < 0.01) reported the largest effects using land-based training.

Moreover, no evidence of publication bias was detected based on the funnel plot and Egger’s test, although the limited number of included studies may reduce the sensitivity of these assessments. Meta-regression analysis did not identify a significant moderating effect of intervention duration (6 to 12 weeks) on the pooled effect size (95% CI: −3.70 to 2.71; *p* = 0.76).

**Fig. 4:**
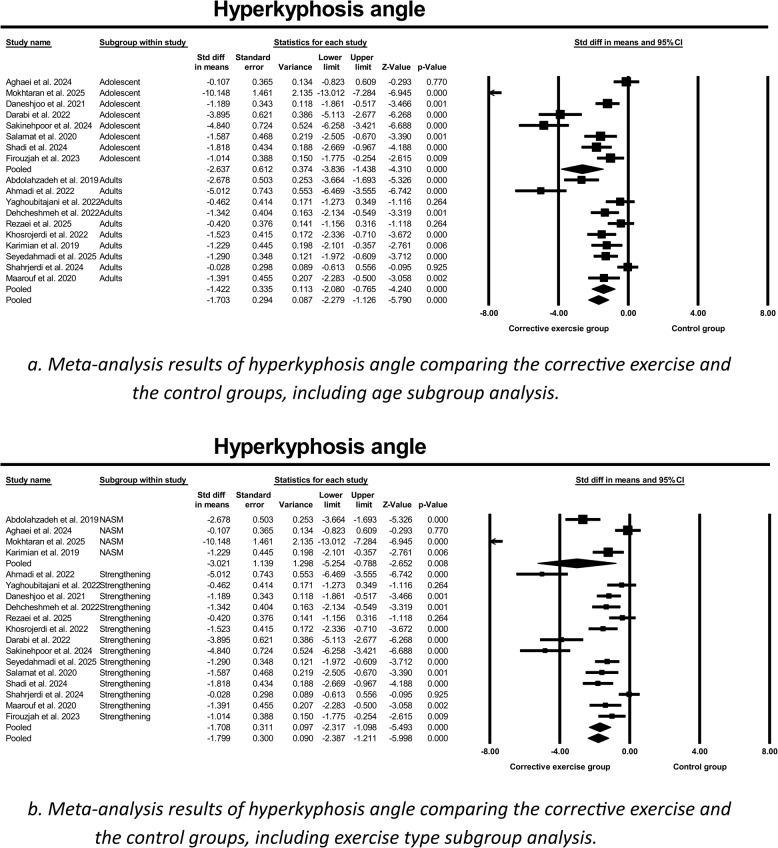
**a**.Meta-analysis results of hyperkyphosis angle comparing the corrective exercise and the control groups, including age subgroup analysis. **b **Meta-analysis results of hyperkyphosis angle comparing the corrective exercise and the control groups, including exercise type subgroup analysis

Eighteen studies investigated the effect of the CE on hyperkyphosis (°), using the flexible ruler [11–13, 27, 29, 31, 32, 34, 35, 37, 39, 40, 43–47, 49]. The results of the meta-analysis suggested strong evidence of a significant decrease in hyperkyphosis in the CE group compared to the control group. Forest plot analysis of the data showed a significant improvement in hyperkyphosis angle in combined adolescent and adults (SMD: −1.70; 95% CI: −2.27 to −1.12; *p* < 0.01) with high heterogeneity (I^2^ = 88%, *p* < 0.01), adolescent (SMD: −2.63; 95% CI: −3.83 to −1.43; *p* < 0.01) with high heterogeneity (I^2^ = 92%, *p* < 0.01), and adults (SMD: −1.42; 95% CI: −2.08 to −0.76; *p* < 0.01) with high heterogeneity (I^2^ = 84%, *p* < 0.01) (Fig. 4a).

Subgroup analysis for the effect of exercise type showed that although NASM and strengthening exercise both are both effective in reducing hyperkyphosis angle. However, NASM retrieved larger effect size (SMD: −3.02; 95% CI: −5.25 to −0.79; *p* < 0.01) with high heterogeneity (I^2^ = 94%, *p* < 0.01) compared to strengthening exercise (SMD: −1.70; 95% CI: −2.31 to −1.09; *p* < 0.01) with high heterogeneity (I^2^ = 86%, *p* < 0.01). But the study by Mokhtaran et al. (2025) has yielded such results. Therefore, these findings warrant cautious interpretation (Fig. 4b).

To compare the effects of aquatic- and land-based CE on hyperkyphosis angle, only Ahmadi et al. 2022 (SMD: −5.01; 95% CI: −6.46 to −3.55; *p* < 0.01) implemented aquatic-based CE. In contrast, Mokhtaran et al. (2025) reported the largest effect with land-based training (SMD: −10.14; 95% CI: −13.01 to −7.28; *p* < 0.01).

**Fig. 5:**
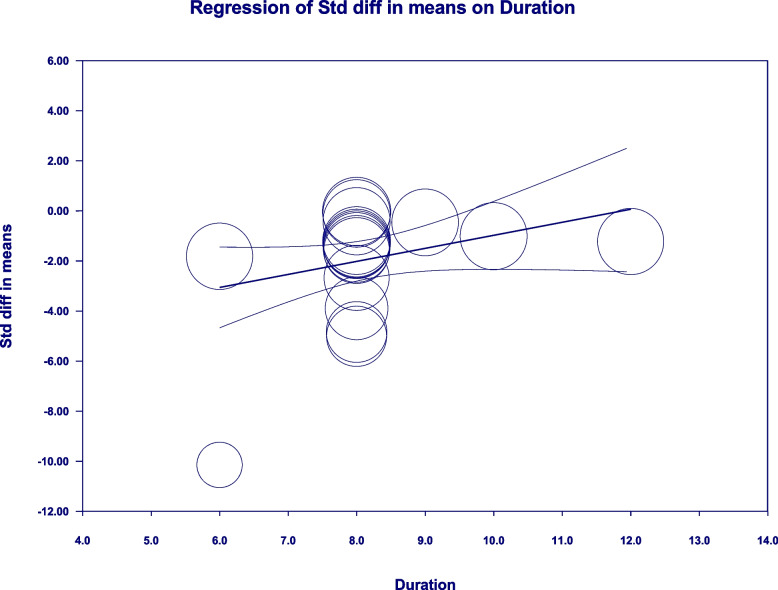
Random-effects meta-regression of effect size on intervention duration. The fitted regression line indicates a statistically significant association

Moreover, no evidence of publication bias was detected based on the funnel plot and Egger’s test, although the limited number of included studies may reduce the sensitivity of these assessments. Meta-regression analysis revealed that the duration of the intervention (6 to 12 weeks) was inversely associated with the pooled effect size (95% CI: −10.39 to −1.96; *p* < 0.01; z = −2.87) (Fig. 5).

### Function

**Fig. 6:**
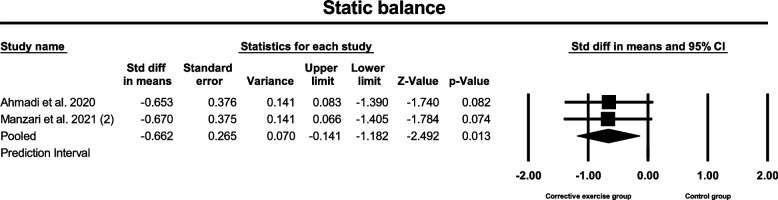
Results of the meta-analysis of static balance error between the corrective exercise and the control groups

**Fig. 7:**
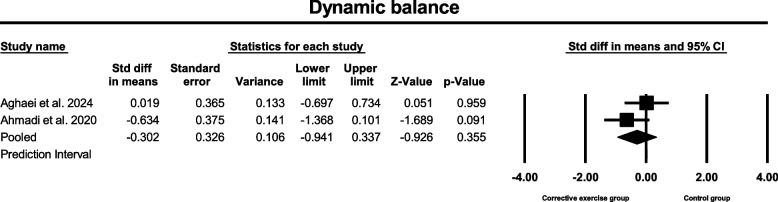
Results of the meta-analysis of dynamic balance error between the corrective exercise and the control groups

Three studies investigated the effect of the CE on balance, using the upper limb Y-balance test (cm) [13, 42] and the Biodex (°) [28]. The results of the meta-analysis suggested moderate evidence of a significant decrease in static balance error (SMD: −0.66; 95% CI: −1.18 to −0.14; *p* < 0.05) with no heterogeneity (I^2^ = 0%, *p* = 0.97) (Fig. 6) in the CE group compared to the control group. Additionally, conflicting evidence suggested no significant changes in dynamic balance error (SMD: −0.30; 95% CI: −0.94 to 0.33; *p* = 0.35) with low to moderate heterogeneity (I^2^ = 35%, *p* = 0.21) (Fig. 7). Formal assessment of publication bias was not feasible because of the limited number of studies in each subgroup.

**Fig. 8:**
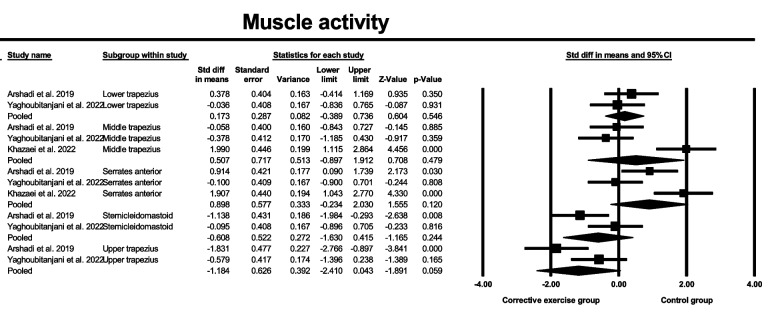
Results of the meta-analysis of scapula muscle activity between the corrective exercise and the control groups

Three studies investigated the effect of the CE on muscle activation (%MVIC), using EMG [11, 12, 38]. The results of the meta-analysis suggested moderate evidence with no significant changes in upper trapezius muscle (SMD: −1.18; 95% CI: −2.41 to 0.04; *p* = 0.06) with high heterogeneity (I^2^ = 72%, *p* = 0.06), lower trapezius muscle (SMD: 0.17; 95% CI: −0.38 to 0.73; *p* = 0.54) with no heterogeneity (I^2^ = 0%, *p* = 0.54), and sternocleidomastoid muscle (SMD: −0.60; 95% CI: −1.63 to 0.41; *p* = 0.24) with moderate to high heterogeneity (I^2^ = 65%, *p* = 0.09) in the CE group compared to the control group. Additionally, meta-analysis showed conflicting evidence in the middle trapezius muscle (SMD: 0.50; 95% CI: −0.85 to 1.92; *p* = 0.48) with high heterogeneity (I^2^ = 88%, *p* < 0.01), serratus anterior muscle (SMD: 0.89; 95% CI: −0.23 to 2.03; *p* = 0.12) with high heterogeneity (I^2^ = 81%, *p* < 0.01) (Fig. 8). Formal assessment of publication bias was not feasible because of the limited number of studies in each subgroup.

**Fig. 9:**
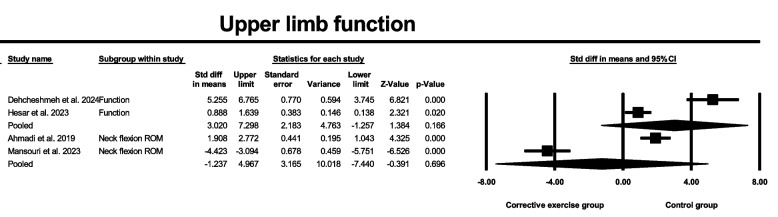
Results of the meta-analysis of function between the corrective exercise and the control groups

Four studies investigated the effect of the CE on upper limb function, using the upper limb Y-balance test (cm) [33, 36] and ROM of neck flexion (°) [10, 30]. The results of the meta-analysis suggested conflicting evidence with no significant changes in upper limb function (SMD: 3.02; 95% CI: −1.25 to 7.29; *p* = 0.16) with high heterogeneity (I^2^ = 96%, *p* < 0.01), and neck flexion ROM (SMD: −1.23; 95% CI: −7.44 to 4.96; *p* = 0.69) with high heterogeneity (I^2^ = 98%, *p* < 0.01) (Fig. 9). Formal assessment of publication bias was not feasible because of the limited number of studies in each subgroup.

### Pain

**Fig. 10:**
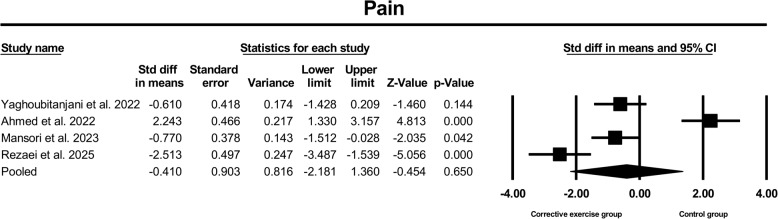
Results of the meta-analysis of pain between the corrective exercise and the control groups

Four studies investigated the effect of the CE on neck pain, using VAS, Numeric Pain Rating Scale, and pain ruler (scale) [10, 11, 46, 50]. The results of the meta-analysis suggested conflicting evidence with no significant changes in pain (SMD: −0.41; 95% CI: −2.18 to 1.36; *p* = 0.65) with high heterogeneity (I^2^ = 94%, *p* < 0.01) (Fig. 10). Formal assessment of publication bias was not feasible because of the limited number of studies in each subgroup.

## Discussion

This study systematically reviewed the impact of the CE on posture, function, and pain in individuals who have UCS based on evidence from RCTs. We hypothesized that the CE would significantly enhance alignment, improve faulty muscle activation, restore function and balance, and reduce pain in these populations.

### Summary of main findings

Overall, the results showed a clear divide: CE produced strong and consistent improvements in postural measures, while its effects on function, pain, balance, and muscle activation were not significant. Across 28 studies and 901 participants, posture-related outcomes, including forward head, forward shoulder, and hyperkyphosis angles, demonstrated significant and large effect sizes favoring CE. In contrast, outcomes related to balance, muscle activation, upper limb function, cervical ROM, and pain showed weak, conflicting, or clinically untrustworthy findings, primarily due to extremely high heterogeneity, differences in study design, and short-term interventions. These results indicate that CE is highly effective for structural alignment but less consistent in producing functional improvements or pain relief.

### Comparison with prior meta-analyses and novelty

Our findings are broadly consistent with prior meta-analyses that reported improvements in postural measures following corrective or posture-focused exercises. However, earlier reviews often included fewer RCTs, focused on isolated outcomes such as forward head posture or neck pain, and rarely assessed multiple functional domains.

In contrast, our review included 28 RCTs with 901 participants and synthesized both structural (posture) and functional outcomes (balance, muscle activation, upper limb function, ROM, and pain). We found that while CE reliably improves postural alignment, its effects on functional outcomes and pain were not significant.

High-quality RCTs employing active rehabilitation interventions, such as scapular stabilization combined with dynamic neuromuscular or respiratory-integrated training, have shown more robust improvements in posture, muscle activation, and related clinical outcomes. For instance, adding respiratory training to scapular stabilization resulted in greater improvements in alignment and shoulder girdle muscle activation compared with stabilization alone in adolescents with UCS [51]. Similarly, combined stabilization-based protocols demonstrated favorable changes in posture and associated muscle activation, highlighting the potential for integrated neuromuscular control to enhance functional outcome [52]. These comparisons underscore that while CE is highly effective for structural correction, integrating stabilization and dynamic neuromuscular functional training may be necessary to achieve broader improvements in pain and functional performance.

### Primary outcomes

The meta-analysis provides strong evidence that CE produces significant improvements in forward head angle, forward shoulder angle, and hyperkyphosis angle among individuals with upper crossed syndrome (UCS). The certainty of this evidence was rated as moderate, reflecting serious concerns regarding risk of bias and very serious concerns related to inconsistency across studies. Despite substantial heterogeneity, the large effect sizes indicate that CE, by restoring muscular balance and correcting dysfunctional movement patterns, effectively enhances postural alignment.

Postural abnormalities, including forward head and shoulders, and increased thoracic kyphosis, are primarily driven by weakness and elongation of deep cervical flexors, upper thoracic extensors, and scapular stabilizers, combined with shortening of anterior shoulder muscles [7]. Interventions targeting the strengthening of weakened muscles and stretching of shortened muscles appear to effectively mitigate these postural dysfunctions [9].

The absence of statistical significance in the NASM subgroup in the forward shoulder angle is most likely attributable to the inclusion of only three studies, a limitation that substantially reduces statistical power and renders the pooled estimate highly sensitive to individual trial characteristics. One study included children, introducing developmental variability in neuromuscular control and postural adaptation that may have influenced the overall effect [13]. Moreover, the phased structure of the NASM approach, progressing from inhibition and lengthening to activation and integration [53], may distribute the training stimulus across multiple objectives, thereby providing a less concentrated strengthening load on the scapular retractors compared with targeted single-phase resistance-based CEs. Collectively, these factors indicate that the non-significant result more likely reflects limited and heterogeneous evidence than a definitive lack of effectiveness.

Variability in outcomes across studies reflects differences in participant characteristics, such as age, occupation, and baseline postural severity, as well as the predominance of short-term follow-up periods. Moreover, most studies focused on static postural measures, with limited assessment of functional performance, sensorimotor control, or psychosocial factors [10–13, 27–45].

Intervention duration may also influence hyperkyphosis outcomes, with some analyses suggesting greater improvements following 6-week interventions compared with 12-week programs. This seemingly counterintuitive result may be explained by early neuromuscular adaptations during the initial weeks of CE, as well as potential differences in exercise intensity, adherence, or participant fatigue over longer interventions. Notably, the 6-week study by Mokhtaran et al., which reported an unusually large effect size, may have disproportionately influenced meta-regression results. Therefore, these findings should be interpreted with caution, as they may reflect study-level variability rather than a true causal effect of intervention duration.

Only one study investigated aquatic-based CE, preventing formal subgroup analysis by training environment. Nonetheless, descriptive comparisons suggest that aquatic-based CE may confer substantial postural improvements, although similar or even greater effect sizes were observed in some land-based programs. Future studies incorporating multiple aquatic-based trials are necessary to enable robust subgroup analyses and to clarify potential differences between aquatic and land-based CE interventions.

These findings underscore the potential of CE as a targeted intervention for postural correction, while highlighting the need for standardized protocols and higher-quality trials to confirm its efficacy.

### Secondary outcome

#### Balance

The meta-analysis indicates that CE significantly improves static balance error in individuals with upper crossed syndrome (UCS), with high-certainty evidence supported by studies of moderate to good methodological quality and generally low risk of bias. In contrast, evidence for dynamic balance was not significant and of low certainty, high risk of bias, imprecision, and variability across studies. This discrepancy likely stems from the predominant focus on static CE programs aimed at postural alignment, rather than task-specific dynamic or neuromuscular training.

Disruption of normal postural alignment in UCS can prevent body segments from maintaining their optimal anatomical positioning, potentially displacing the center of gravity [54]. However, the extent to which these alterations affect dynamic stability remains debated. Most included studies focused primarily on static CE targeting postural alignment, without incorporating dynamic or neuromuscular balance training [10–13, 27–45].

Consequently, while static CE reliably enhances static balance, improving dynamic balance likely requires targeted interventions incorporating proprioception, functional movement patterns, and neuromuscular control strategies. Future research should prioritize high-quality, well-controlled trials that integrate both static and dynamic components to fully evaluate the potential of CE in balance rehabilitation for UCS.

### Muscle activity

The analysis of CE reveals largely non-significant and limited effects on muscle activation patterns in upper crossed syndrome, with evidence certainty varying by muscle and consistent methodological limitations across studies. Moderate-certainty evidence indicates minimal changes in upper and lower trapezius activation, tempered by high risk of bias and imprecision, while low-certainty evidence suggests some degree of sternocleidomastoid activation, constrained by high risk of bias, imprecision, and inconsistency. Evidence for the middle trapezius and serratus anterior is conflicting and of very-low to low certainty, similarly, undermined by high risk of bias, imprecision, and inconsistency. These modest effects may stem from insufficient intervention dosage and short exposure periods, typically 6 to 12 weeks, which is likely inadequate for achieving sustained neuromuscular re-education or structural postural adaptation in chronic upper crossed syndrome, as well as considerable variability in study design [52, 55]. Marked heterogeneity in protocols, participant characteristics, and measurement methods further limits interpretability and generalisability [56].

In line with Janda’s muscle imbalance approach, the sternocleidomastoid and upper trapezius are considered tonic muscles that tend to become overactive and therefore require inhibition, whereas the middle and lower trapezius and serratus anterior are phasic muscles that are prone to inhibition and should be facilitated [28]. Although our findings demonstrated changes in this expected direction, these differences did not reach statistical significance, suggesting a potential neuromuscular rebalancing effect that may not have been sufficiently strong to produce significant between-group differences [51].

When compared with alternative exercise interventions, CE appears less effective in eliciting robust muscle activation. Targeted approaches, such as elevation protocols for the upper and lower trapezius or wall slides for the serratus anterior, have demonstrated greater engagement of these muscles [57]. Systematic reviews indicate that specific periscapular exercises more effectively optimize muscle ratios than general CEs, and push-up variations on unstable surfaces can substantially increase lower trapezius activation. These findings suggest that incorporating task-specific movements, instability, or progressive loading may enhance neuromuscular outcomes beyond what CE alone can achieve, highlighting the need for more tailored intervention strategies. These comparisons suggest that incorporating elements like instability or specific movement patterns might amplify outcomes beyond what CE alone achieves [52].

### Upper limb function

CE demonstrated no significant change in upper limb function, with low certainty due to high risk of bias, imprecision, and inconsistency. These outcomes, measured via Y-Balance Tests (YBT) and neck range of motion (ROM), likely reflect high heterogeneity across studies stemming from differences in evaluation tools, intervention protocols, and participant characteristics. This suggests that CE alone may be insufficient to produce reliable functional improvements and may require supplementation with targeted or multimodal approaches.

For YBT outcomes, no established minimal clinically important difference (MCID) currently exists. Therefore, minimal detectable change (MDC) values were used for reference, with UQ-YBT MDC95% reported between approximately 4.8–21.1% of normalized reach distance, representing the smallest change beyond measurement error [58]. In the included studies, observed SMDs fell below this MDC range, indicating that although some improvements were statistically significant, they may not be clinically meaningful.

By comparison, alternative interventions appear more effective. Exercise therapy has demonstrated superiority over controls in managing chronic neck pain, while combining stabilization exercises with manual therapy reduces disability more effectively than stabilization alone [59]**.** Virtual reality–based programs can enhance mobility and alleviate pain in upper limb rehabilitation, and ergonomic or robotic-assisted interventions offer promising results for neck and upper extremity disorders [60]. These findings highlight that multimodal or technology-integrated strategies may outperform standalone CE in improving functional outcomes.

Successful implementation of CE requires precise exercise execution with optimal alignment, along with comprehensive participant education. Modifications to the FITT principle (Frequency, Intensity, Time, and Type) should follow established guidelines such as those from the American College of Sports Medicine (ACSM) [61]. Additionally, Lederman’s framework underscores the importance of body awareness; without it, individuals may fail to engage the correct muscles and movement patterns, potentially limiting progress or exacerbating dysfunction [62]. Variations in exercise environments, including aquatic settings in some studies [28–30], introduce further confounding factors that may contribute to inconsistent results.

### Pain

The meta-analysis revealed no significant reduction in pain in the CE group compared to controls, supported by low-certainty evidence due to high risk of bias, inconsistency, and imprecision**.** These findings suggest that exercise-based interventions alone may have limited effectiveness for pain management in individuals with upper crossed syndrome. It is important to note that pain is a multifactorial outcome, influenced by biomechanical, neurological, and psychosocial factors, and may not be adequately addressed through exercise alone, particularly when pain is not the primary target of intervention. For context, the Minimal Clinically Important Difference (MCID) for pain has been reported as 10–20 mm for VAS and 1–2 points for the numerical rating scale [63], highlighting that even statistically significant changes may not always translate to meaningful clinical improvement.

In the early stages, postural syndromes such as UCS are frequently asymptomatic; however, prolonged postural deviations may progressively increase mechanical loading on the cervical and upper thoracic regions, ultimately contributing to the development of pain and functional limitations [52]. Consequently, the absence of significant pain reduction may be partly explained by low baseline pain levels among participants and the indirect nature of exercise effects on pain modulation.

### Limitations related to the included studies

Except for a limited number of studies that assessed the sustainability of intervention outcomes, most included trials lacked follow-up periods, thereby restricting conclusions regarding the long-term efficacy of exercise-based corrective interventions [10–13, 27, 31–45].

Additionally, the included studies involved heterogeneous age groups and applied diverse exercise protocols, many of which were primarily strength-oriented and lacked a functional or task-specific focus. Although some studies briefly addressed functional outcomes, the overall emphasis of the interventions was predominantly structural rather than holistic or movement-centered.

Furthermore, substantial heterogeneity was observed across studies, particularly in primary outcome measures. This heterogeneity must be interpreted carefully, as it diminishes confidence in the pooled effect sizes and reflects variability in participant characteristics, intervention duration/intensity, and study methodology. Another limitation was the heterogeneity of control group conditions. Control groups included continuation of regular athletic training, usual daily activities, vestibular exercise, monthly health education, and in many cases no structured intervention. This variability may have contributed to heterogeneity across the pooled analysis. Variability in risk of bias and the potential influence of publication bias further constrain the robustness of the evidence.

### Limitations of the review itself

The review did not search grey literature sources, which may have resulted in the omission of unpublished or non-peer-reviewed studies. These limitations may have contributed to publication bias and should be considered when interpreting the findings.

Collectively, these limitations warrant cautious interpretation of the results, as the current evidence base remains inconsistent despite some positive trends. Future research should adopt a more functionally oriented perspective, prioritize subject-centered and performance-based outcomes, and examine the effects of comprehensive CE frameworks (e.g., NASM- or Sahrmann-based approaches) rather than isolated strengthening interventions alone. Incorporating neuromuscular training elements that emphasize proprioceptive control and functional movement patterns may further enhance clinical relevance and long-term outcomes.

## Conclusions

In conclusion, this systematic review and meta-analysis of 28 RCTs involving 901 UCS patients demonstrates that CE yields strong, consistent improvements in postural alignment (forward head, shoulder, and hyperkyphosis angle) with large effect sizes. However, these large effect sizes should be interpreted with caution due to the very high heterogeneity across studies. Effects on function, balance, muscle activation, ROM, and pain were not significant and unreliable due to high heterogeneity and limited protocols.

Clinically, CE is highly effective for structural correction, but our results indicate inconsistent improvements in functional outcomes, pain, balance, and muscle activation. Comparisons with high-quality RCTs suggest that interventions combining stabilization, dynamic neuromuscular, or respiratory-integrated training produce larger functional and pain-related benefits. Therefore, integrating CE with such complementary therapies may enhance overall outcomes. Future research should investigate longitudinal, multimodal, scapula-focused, and functionally oriented programs to optimize UCS management.

## Supplementary Information

Supplementary Material 1. The GRADE.

Supplementary Material 2. Raw data UCS.

Supplementary Material 3. Prisma Checklist.

## Data Availability

Detailed search results are available on request (Jalilimostafa3@gmail.com).

## Abbreviation

UCS: Upper Crossed Syndrome
CE: Corrective Exercises
RCTs: Randomized Controlled Trials
SMD: Standardized Mean Differences
CI: Confidence Intervals
NASM: National Academy of Sports Medicine
VAS: Visual Analog Scale
sEMG: Surface Electromyography
ROM: Range Of Motion
CMA: Comprehensive Meta-Analysis software
NR: Not Reported
CVA: Craniovertebral angle
FITT: Frequency, Intensity, Time, and Type
ACSM: American College of Sports Medicine
MJB: Mostafa Jalili Bafrouei
FK: Fateme Khorramroo
MR: Mohamad Rostami
