# Supplementary material for: Corrective exercises strongly improve posture but fail to produce consistent clinical or functional benefits in patients with upper crossed syndrome: a systematic review and meta-analysis of randomized controlled trials
Source: BMC Sports Sci Med Rehabil. 2026 May 28;18:302. doi: 10.1186/s13102-026-01707-8 (PMC13326462; doi:10.1186/s13102-026-01707-8)
Supplement: Supplementary file 1 — Supplementary Material 1. The GRADE. [file 13102_2026_1707_MOESM1_ESM.docx]

| **№ of studies** | **Study design** | | **Risk of bias** | | **Inconsistency** | **Indirectness** | **Imprecision** | | **Other considerations** | | **Absolute (95% CI)** | | **Certainty** | |
| --- | --- | --- | --- | --- | --- | --- | --- | --- | --- | --- | --- | --- | --- | --- |
| **Forward head angle (follow-up: range 6 weeks to 12 weeks)** | | | | | | | | | |  |  |  |  |  |
| **18** | **randomized trials** | | **serious** | | **very serious** | **not serious** | **not serious** | | **strong association, all plausible residual confounding words reduce the demonstrated effect** | | **SMD 1.49 SD lower (1.91 lower to 1.06 fewer)** | | **⨁⨁⨁◯ Moderate** | |
| **Forward shoulder angle (follow-up: range 6 weeks to 12 weeks)** | | | | | | | | | |  |  |  |  |  |
| **18** | **randomized trials** | | **serious** | | **very serious** | **not serious** | **not serious** | | **strong association, all plausible residual confounding words reduce the demonstrated effect** | | **SMD 1.53 SD lower (1.96 lower to 1.09 fewer)** | | **⨁⨁⨁◯ Moderate** | |
| **Static balance error (follow-up: range 6 weeks to 12 weeks)** | | | | | | | | | |  |  |  |  |  |
| **2** | **randomized trials** | | **serious** | | **not serious** | **not serious** | **not serious** | | **all plausible residual confounding words reduce the demonstrated effect** | | **SMD 0.66 SD lower (1.18 lower to 0.14 fewer)** | | **⨁⨁⨁⨁ High** | |
| **Dynamic balance error (follow-up: range 6 weeks to 12 weeks)** | | | | | | | | | |  |  |  |  |  |
| **2** | **randomized trials** | | **serious** | | **serious** | **not serious** | **serious** | | **all plausible residual confounding words reduce the demonstrated effect** | | **SMD 0.30 SD lower** **(0.94 lower to 0.33 higher)** | | **⨁⨁◯◯ Low** | |
| **Hyperkyphosis angle (follow-up: range 6 weeks to 12 weeks)** | | | | | | | | | |  |  |  |  |  |
| **18** | **randomized trials** | | **serious** | | **very serious** | **not serious** | **not serious** | | **strong association, all plausible residual confounding words reduce the demonstrated effect** | | **SMD 1.70 SD lower (2.27 lower to 1.12 fewer)** | | **⨁⨁⨁◯ Moderate** | |
| **Upper trapezius muscle (follow-up: range 6 weeks to 12 weeks)** | | | | | | | | | |  |  |  |  |  |
| **2** | **randomized trials** | | **serious** | | **very serious** | **not serious** | **serious** | | **strong association, all plausible residual confounding words reduce the demonstrated effect** | | **SMD 1.18 SD lower (2.41 lower to 0.04 higher)** | | **⨁⨁⨁◯ Moderate** | |
| **Lower trapezius muscle (follow-up: range 6 weeks to 12 weeks)** | | | | | | | | | |  |  |  |  |  |
| **2** | **randomized trials** | | **serious** | | **not serious** | **not serious** | **serious** | | **all plausible residual confounding words reduce the demonstrated effect** | | **SMD 0.17 SD lower (0.38 lower to 0.73 higher)** | | **⨁⨁⨁◯ Moderate** | |
| **Sternocleidomastoid muscle (follow-up: range 6 weeks to 12 weeks)** | | | | | | | | | |  |  |  |  |  |
| **2** | **randomized trials** | | **serious** | | **serious** | **not serious** | **serious** | | **all plausible residual confounding words reduce the demonstrated effect** | | **SMD 0.60 SD lower (1.63 lower to 0.41 higher)** | | **⨁⨁◯◯ Low** | |
| **Middle trapezius muscle (follow-up: range 6 weeks to 12 weeks)** | | | | | | | | | |  |  |  |  |  |
| **3** | **randomized trials** | | **serious** | | **very serious** | **not serious** | **serious** | | **all plausible residual confounding words reduce the demonstrated effect** | | **SMD 0.50 SD lower (0.85 lower to 1.92 higher)** | | **⨁◯◯◯ Very low** | |
| **Serratus anterior muscle (follow-up: range 6 weeks to 12 weeks)** | | | | | | | | | |  |  |  |  |  |
| **3** | **randomized trials** | | **serious** | | **very serious** | **not serious** | **serious** | | **Strong association, all plausible residual confounding words reduce the demonstrated effect** | | **SMD 0.89 SD lower (0.23 lower to 2.03 higher)** | | **⨁⨁◯◯ Low** | |
| **Pain (follow-up: range 6 weeks to 12 weeks)** | | | | | | | | | |  |  |  |  |  |
| **4** | | **randomized trials** | | **serious** | **very serious** | **not serious** | | **serious** | **all plausible residual confounding words reduce the demonstrated effect** | | **SMD 0.41 SD lower (2.18 lower to1.36 higher)** | **⨁◯◯◯ Very low** | |  |

**Upper limb function (follow-up: range 6 weeks to 12 weeks)**

| **3** | **randomized trials** | **serious** | **very serious** | **not serious** | **serious** | **Strong association, all plausible residual confounding words reduce the demonstrated effect** | **SMD 3.02 SD lower (1.25 lower to7.29 higher)** | **⨁⨁◯◯ Low** |
| --- | --- | --- | --- | --- | --- | --- | --- | --- |

**Neck flexion ROM (follow-up: range 6 weeks to 12 weeks)**

| **3** | **randomized trials** | **serious** | **very serious** | **not serious** | **serious** | **Strong association, all plausible residual confounding words reduce the demonstrated effect** | **SMD 1.23 SD lower (7.44 lower to4.96 higher)** | **⨁⨁◯◯ Low** |
| --- | --- | --- | --- | --- | --- | --- | --- | --- |
